# Targeting valine catabolism to inhibit metabolic reprogramming in prostate cancer

**DOI:** 10.1038/s41419-024-06893-2

**Published:** 2024-07-18

**Authors:** Charles L. Bidgood, Lisa K. Philp, Anja Rockstroh, Melanie Lehman, Colleen C. Nelson, Martin C. Sadowski, Jennifer H. Gunter

**Affiliations:** 1 Queensland University of Technology (QUT), Australian Prostate Cancer Research Centre - Queensland, Centre for Genomics and Personalised Health, School of Biomedical Sciences, Translational Research Institute, Brisbane, QLD Australia; 2 University of British Columbia, Vancouver Prostate Centre, Department of Urologic Sciences, Vancouver, BC Canada; 3 University of Bern, Institute for Tissue Medicine and Pathology, Bern, Switzerland

**Keywords:** Cancer metabolism, Prostate cancer

## Abstract

Metabolic reprogramming and energetic rewiring are hallmarks of cancer that fuel disease progression and facilitate therapy evasion. The remodelling of oxidative phosphorylation and enhanced lipogenesis have previously been characterised as key metabolic features of prostate cancer (PCa). Recently, succinate-dependent mitochondrial reprogramming was identified in high-grade prostate tumours, as well as upregulation of the enzymes associated with branched-chain amino acid (BCAA) catabolism. In this study, we hypothesised that the degradation of the BCAAs, particularly valine, may play a critical role in anapleurotic refuelling of the mitochondrial succinate pool, as well as the maintenance of intracellular lipid metabolism. Through the suppression of BCAA availability, we report significantly reduced lipid content, strongly indicating that BCAAs are important lipogenic fuels in PCa. This work also uncovered a novel compensatory mechanism, whereby fatty acid uptake is increased in response to extracellular valine deprivation. Inhibition of valine degradation via suppression of 3-hydroxyisobutyryl-CoA hydrolase (HIBCH) resulted in a selective reduction of malignant prostate cell proliferation, decreased intracellular succinate and impaired cellular respiration. In combination with a comprehensive multi-omic investigation that incorporates next-generation sequencing, metabolomics, and high-content quantitative single-cell imaging, our work highlights a novel therapeutic target for selective inhibition of metabolic reprogramming in PCa.

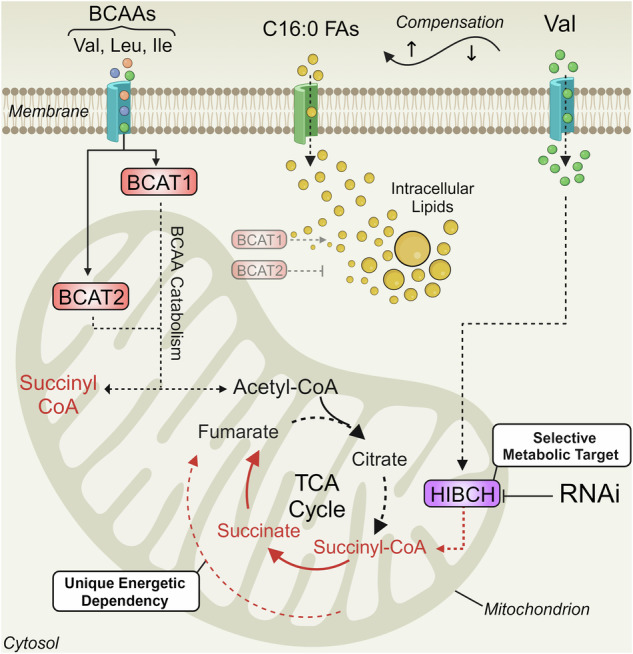

## Introduction

Metastatic castration-resistant prostate cancer (mCRPC) is the second leading cause of male cancer mortality, resulting in more than 375,000 deaths globally each year [1]. The objective of the current standard-of-care therapies is to inhibit the androgen receptor (AR) signalling axis, a major driver of tumour growth and disease progression [2]. Restoration of AR signalling despite castrate levels of androgens drives resistance to these therapies, ultimately resulting in treatment failure. The development of therapeutic resistance is underpinned by fundamental alterations to cellular energetics in response to the heightened metabolic demand experienced by the tumour [3–5]. Many studies have previously described dysregulated lipid metabolism as a core metabolic alteration in prostatic malignancy [6–9]. The remodelling of oxidative phosphorylation (OxPhos) has also recently been identified as a key characteristic of high-grade prostate cancer tissue. These studies report a metabolic shift from NADH-linked respiration to succinate-linked respiratory function (SLRF) [10, 11]. Increased SLRF in high-grade PCa tissue is also coincident with a heightened burden of heteroplasmic mitochondrial DNA (mtDNA) mutations [12].

The branched-chain amino acids (BCAAs), leucine, isoleucine and valine have been shown to contribute to multiple pro-oncogenic processes in PCa [13–16], but most of what we know about their catabolism comes from their established role as carbon donors during adipogenesis [17]. The complete catabolism of the BCAAs produce two major mitochondrial intermediates for tricarboxylic acid (TCA) cycle replenishment: acetyl-CoA and succinyl-CoA. BCAA catabolism is an important fuel source for adipogenesis, demonstrated by significant isotopic carbon labelling (~40%) of acetyl-CoA from the BCAAs in matured adipocytes [17]. Whether the BCAAs have a similar role in PCa, however, still remains to be elucidated. Given the importance of both acetyl-CoA for lipid homeostasis and succinyl-CoA for maintaining SLRF, the BCAA catabolic pathway presents a potentially opportune therapeutic target against PCa. The catabolism of all three BCAAs is universally initiated via the branched-chain amino acid aminotransferases (BCAT1 and BCAT2), but most subsequent downstream reactions are amino acid-specific. While BCAA catabolism has been identified as a potential target in multiple cancers, their therapeutic potential remains to be thoroughly investigated.

Here, we report that lipid uptake and content of non-malignant prostate cells exhibit a high metabolic dependency on leucine, and that this dependency switches to valine in malignant PCa cell lines. Given the discovery and the importance of succinate metabolism in PCa, we have further investigated the importance of valine catabolism. From the BCAAs, only valine and isoleucine contribute to succinyl-CoA, which supplies succinate in the TCA cycle. 3-hydroxyisobutyryl-CoA hydrolase (HIBCH) converts 3-hydroxyisobutyryl-CoA (3HIB-CoA) to 3-hydroxyisobutyric acid (3HIB) and is a central enzyme of valine catabolism. In models of colorectal cancer, disruption of valine catabolism via the inhibition of HIBCH reduces TCA metabolite levels, inhibits tumour xenograft growth and reduces the occurrence of drug-acquired resistance to bevacizumab [18]. In our study, we have discovered that the BCAAs, including valine, contribute to the lipogenic phenotype of PCa. Furthermore, we demonstrate that metabolic reprogramming of mitochondrial respiration in advanced PCa is associated with increased breakdown of the amino acid valine to replenish the intracellular succinate pool. Disruption of SLRF via inhibition of valine degradation showed promising therapeutic potential and highlights a novel therapeutic approach to combat metabolic reprogramming in PCa.

## Results

### BCAA uptake and catabolism maintains intracellular lipid supply in prostate cancer

**Fig. 1:**
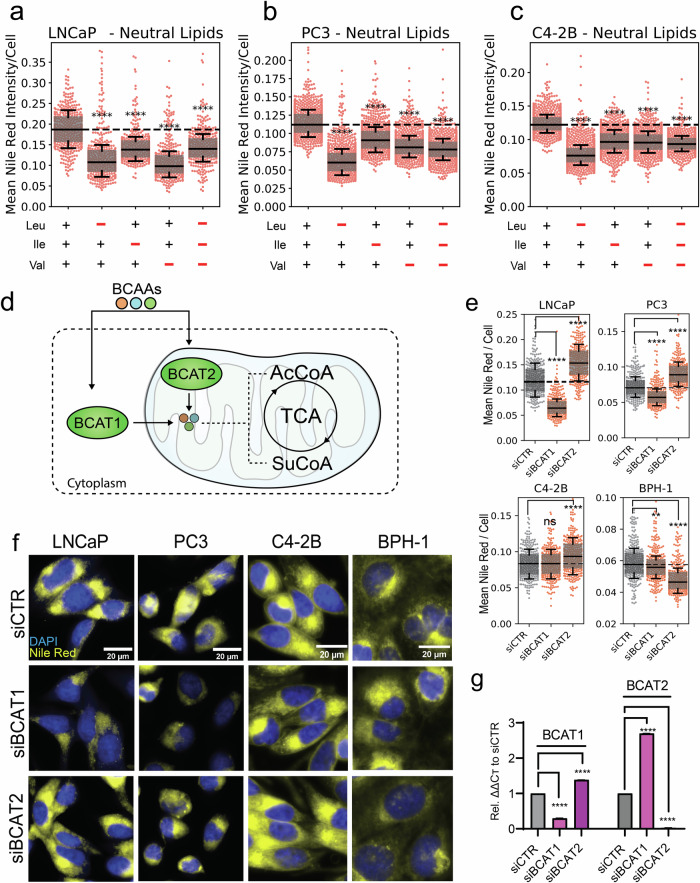
BCAA uptake and catabolism is critical for intracellular lipid maintenance in PCa. **a**–**c** Intracellular neutral lipid content of LNCaP, C4-2B and PC3 PCa cells following 24 h of exogenous BCAA depletion measured by Nile Red staining and quantitative single cell imaging analysis (qSCI), data representative of two independent experiments. **d** Schematic of BCAA catabolism by BCAT1 and BCAT2. **e** qSCI analysis of LNCaP, PC3, C4-2B, and non-malignant BPH-1 cells following Nile Red and DAPI staining after 72 h of siRNA knockdown of either BCAT1 or BCAT2. Data is representative of two independent experiments. **f** Representative fluorescent images of Nile Red staining performed in LNCaP, PC3, C4-2B and BPH-1 cells. **g** mRNA expression of BCAT1 and BCAT2 following 72 h siRNA knockdown (siBCAT1 and siBCAT2) measured by qRT-PCR in LNCaP cells. In all experiments, significance was determined by One-Way ANOVA with Dunnett’s Multiple Comparison Test compared to the vehicle control (+BCAAs or siCTR). Box and whisker plots are shown with their quartile ranges and bar graph error bars represent SEM. ns - not significant, **p < 0.05, ***p < 0.001 ****p < 0.0001.

To evaluate the effects of individual BCAAs on PCa lipid content, a customised BCAA-depleted medium (BDM) was generated to deprive cells of exogenous BCAAs, to which physiological concentrations of leucine, isoleucine, or valine were re-added. This was performed across a spectrum of cell lines representative of multi-stage PCa progression, including androgen-sensitive (LNCaP), androgen-insensitive (C4-2B) and castrate-resistant disease (PC3). LNCaP, C4-2B and PC3 cells were first cultured in BDM for 24 h before being subjected to Nile Red staining. This was performed to quantify intracellular neutral lipid content using our previously established quantitative single-cell imaging (qSCI) analysis pipeline [6]. Preliminary investigations also revealed that 24 h of cell culture in BDM did not result in any observable reductions to cell growth and was thus deemed a suitable timepoint (Supplementary Fig. 1). The absence of all BCAAs resulted in a statistically significant reduction in lipid content within each PCa cell line (Fig. 1a–c) indicating that the BCAAs likely contribute to the PCa lipidome. While leucine starvation elicited the greatest reduction to lipids in C4-2B and PC3 cells, valine deprivation caused a comparable reduction in lipid content in androgen-sensitive LNCaP cells. Total BCAA depletion also did not have an additive effect compared to the depletion of any individual BCAA, suggesting the acquisition of fatty acids via alternative sources. To confirm whether these effects were from BCAA catabolism, independent from their proteinogenic properties, lipid content was again measured following inhibition of BCAA catabolism by small interfering RNA (siRNA) knockdown of *BCAT1* and *BCAT2* (Fig. 1d). In addition to PCa cell lines, we also investigated *BCAT1* and *BCAT2* knockdown in the non-malignant prostatic epithelial cell line BPH-1. Significant reductions in lipid content were observed following *BCAT1* knockdown in LNCaP and PC3 cells, while only minor decreases were observed within BPH-1 cells supporting a potential lipogenic role for BCAAs in PCa cell lines. No significant changes were observed in C4-2B cells (Fig. 1e, f). Surprisingly, *BCAT2* knockdown stimulated the accumulation of lipids within all PCa cell lines, while exerting an inhibitory effect on lipids in the benign BPH-1 cell line. This compensatory phenotype was also observable at the transcriptomic level, shown by inverse expression of *BCAT1/2* in response to the suppression of the other (Fig. 1g)*.* The strong upregulation of BCAT1 mRNA expression in response to BCAT2 siRNA could have potentially caused an overcompensation in BCAA-fuelled lipogenesis.

### Exogenous availability of valine co-regulates long-chain fatty acid uptake in PCa

**Fig. 2:**
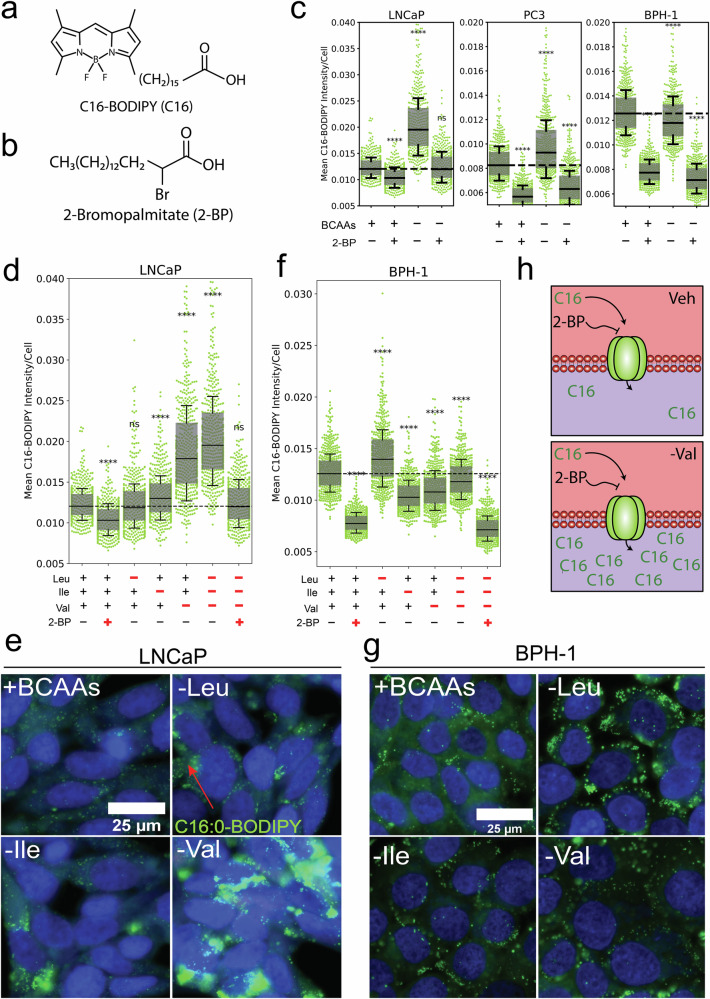
Extracellular valine co-regulates long-chain fatty acid uptake in PCa cells. Chemical structure of (**a**) C16-BODIPY (C16) and (**b**) 2-Bromopalmitate (2-BP). **c**, **d**, **f** Quantitative single cell imaging (qSCI) analysis of C16:0-BODIPY (green) uptake following 24 h of exogenous BCAA (universal and individual) deprivation and/or co-treatment with 2-BP in PCa cell lines LNCaP, PC3 or non-malignant BPH-1 cells. **h** Schematic describing valine’s consequence on C16 uptake in PCa cells and its ability to be inhibited by 2-BP. **e**, **g** Representative live-cell fluorescent images of LNCaP and BPH-1 cell uptake of C16 co-stained with Hoechst 33342 which have been subject to BCAA deprivation. Box and whisker plots show the quartile ranges. Significance determined by One-Way ANOVA with Dunnett’s Multiple Comparison Test compared to the vehicle control (+BCAAs, -2-BP). ns not significant, **p < 0.01, ***p < 0.001, ****p < 0.0001.

Having established an important role for BCAAs in maintaining lipid homeostasis in PCa cells, exogenous fatty acid uptake was explored as a potential compensatory mechanism in response to BCAA starvation. PCa cells were cultured in BDM supplemented with and without individual BCAAs at their physiological concentrations. After 24 h, cells were incubated with the fluorescent palmitate analogue C16:0-BODIPY (C16) (Fig. 2a) to measure long-chain fatty acid uptake in response to BCAA depletion. Co-treatment with the non-fluorescent palmitate analogue 2-bromopalmitate (2-BP), a competitive inhibitor of fatty acid transport (Fig. 2b) was also used to investigate whether this response could be reversed. qSCI analysis revealed that both LNCaP and PC3 cells significantly increased C16 uptake in response to BCAA depletion, while BPH-1 cells significantly reduced C16 uptake (Fig. 2c and Supplementary Fig. 2a–d). Furthermore, co-treatment with 2-BP reduced LNCaP C16 uptake to baseline, and PC3 cell levels below baseline, indicating that C16 uptake was likely fatty acid transporter-mediated (Fig. 2c and Supplementary Fig. 2e). To probe whether this compensatory response was linked to universal BCAA starvation or was induced by a specific BCAA, C16 uptake was measured in the absence of single BCAAs. Our findings report that valine depletion almost exclusively accounted for the compensatory increase in C16 uptake in malignant LNCaP cells (Fig. 2d, e). Inversely, benign BPH-1 cells elicited this response only after leucine deprivation (Fig. 2f, g). Together, these findings highlight an important disparity in substrate dependency between benign and malignant prostate cells, most notably a unique response to valine (Fig. 2h).

### Valine catabolism is associated with enhanced succinate-linked respiratory function, AR activity and survival across the PCa disease spectrum

**Fig. 3:**
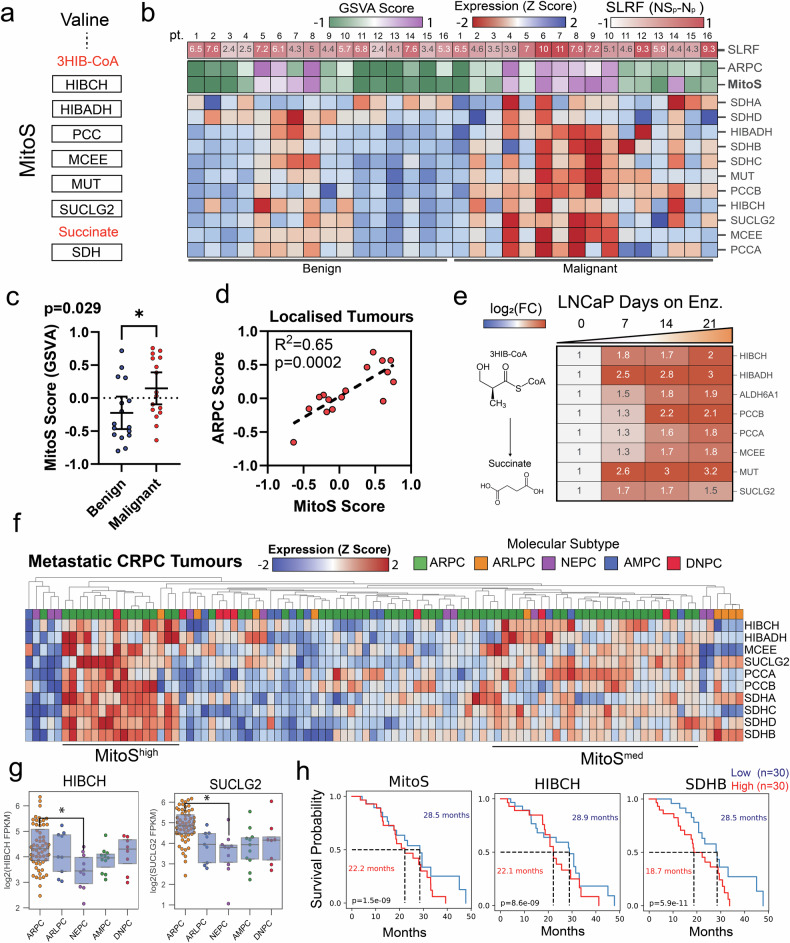
Valine catabolism and succinate-linked respiration is enhanced through PCa progression. **a** MitoS gene signature schematic. **b** Heatmap showing MitoS expression z-scores in benign vs. malignant patient primary prostate tissue with signature scores of MitoS and AR activity (ARPC) as well as functional succinate-oxidation (SLRF). **c** MitoS scoring by gene set variation analysis (GSVA) of benign vs. malignant prostate tissues. Error bars represent SEM. **d** Correlation analysis of MitoS and ARPC score in malignant PCa tissue. **e** Gene expression heatmap of enzymes responsible for 3HIB-CoA to succinate conversion in LNCaP cells treated with 7, 14 and 21 days of 10 µM enzalutamide. **f** Clustered heatmap of MitoS genes in metastatic castrate-resistant PCa (mCRPC) RNAseq stratified by molecular subtype. **g** Gene expression of HIBCH and SUCLG2 across mCRPC subtypes, Box and whisker plots show the quartile ranges. **h** Kaplan–Meier curves displaying the median survival time of mCRPC patients classified into high (*n* = 30, red) or low (*n* = 30, blue) tumour expression of MitoS score, HIBCH and SDHB. Raw datasets obtained from Schopf et al. [12] (**a**–**d**), Tousignant et al. [5] (**f**), Labrecque et al. [19] (**f**, **g**) and Abida et al. [20] (**h**). Significance determined by One-Way ANOVA, *p < 0.05. Tumour subtype: AR-high PCa (ARPC), AR-low PCa (ARLPC), neuroendocrine PCa (NEPC), amphicrine PCa (AMPC) and double-negative AR^-^/NE^-^ PCa (DNPC) as defined by Labrecque et al. [19].

To explore the clinical association of valine catabolism and intratumoural SLRF, publicly available patient-derived datasets were interrogated using a customised metabolic gene signature (denoted as MitoS), incorporating genes encoding enzymes necessary to catabolise 3HIB-CoA into succinate, and the respective subunits of SDH (Fig. 3a). Lending further support to the clinical significance of enhanced MitoS activity linked to valine catabolism, propionyl-CoA carboxylase (*PCCA)*, succinyl-CoA ligase subunit beta (*SUCLG2)* and succinate dehydrogenase subunit C *(SDHC)* were all determined to be within the top 20 most correlated genes in relation to HIBCH expression in a cohort of patients (TCGA-PRAD) with prostate adenocarcinoma (Supplementary Table 3). The MitoS signature was subsequently evaluated against an RNA-seq dataset containing matched benign and malignant tissues from 16 localised PCa prostatectomy samples [12]. The MitoS gene signature was significantly (*p* = 0.029) enriched in malignant versus benign tissue (Fig. 3b, c). Finally, the MitoS signature was compared to a previously published gene signature (ARPC) representing tumours expressing high levels of the AR [19]. Linear regression analysis revealed a statistically significant correlation (*p* = 0.0002) between the MitoS and ARPC gene scores within malignant samples, indicating that valine-supported SLRF is present in localised disease and is highly correlated to AR activity (Fig. 3d). To examine the association of valine catabolism in advanced and metastatic disease, we interrogated a microarray (RNA) dataset generated from long-term (21-day) treatment of PCa cells with the AR antagonist, enzalutamide [5]. This analysis revealed significantly increased levels of the genes responsible for succinate generation downstream of HIBCH in androgen-sensitive LNCaP cells, suggesting that advanced PCa responds to AR antagonism by further upregulating valine catabolism and SLRF (Fig. 3e). To determine the relevance of this mitochondrial phenotype in metastatic castrate-resistant disease, the MitoS signature was compared against a published RNAseq dataset, of molecularly subtyped patient tumours which we then stratified for MitoS enrichment [19]. This uncovered a select cohort of AR positive (ARPC) mCRPC patients whose tumours were both highly (MitoS^high^) and moderately (MitoS^med^) enriched for the MitoS gene signature (Fig. 3f). Inversely, tumours that possessed neuroendocrine PCa features (NEPC) were negatively associated with these genes, notably *HIBCH* and *SUCLG2* (Fig. 3g). Finally, RNAseq of patient tumours with mCRPC (*n* = 208) were analysed against longitudinal survival data [20] to determine whether the MitoS gene signature was predictive of clinical patient outcomes. This analysis revealed that patients with a high MitoS score (top ~15%) were associated with a 6.3 month reduced mean survival time (MST) (*p* < 0.0001) than those in the bottom ~15%. In concordance with this result, analysis of only *HIBCH* demonstrated a 6.8-month reduced MST (*p* < 0.0001), and most notably, the succinate dehydrogenase subunit *SDHB* had a 9.8-month reduced MST (Fig. 3h).

### Inhibition of valine catabolism via HIBCH suppresses prostate cancer metabolism via succinate production

**Fig. 4:**
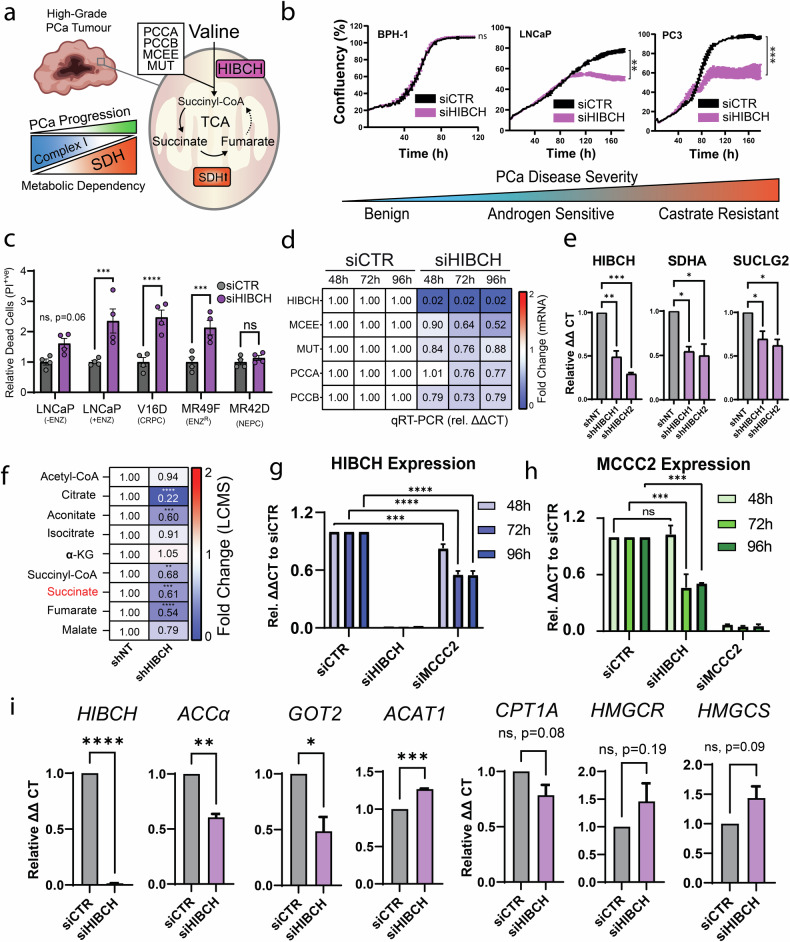
Targeting valine catabolism to inhibit proliferation and metabolic plasticity in PCa. **a** Schematic describing proposed hypothesis that mitochondrial energy remodelling (enhanced succinate oxidation by SDH) is facilitated by enhanced valine catabolism and 3-hydroxyisobutyryl-CoA Hydrolase (HIBCH) activity. **b** Cell confluency time course assay in LNCaP, PC3 and BPH-1 cells, following siRNA transfection of either siCTR or siHIBCH. **c** Live dead analysis of LNCaP±10 µM enzalutamide, V16D, enzalutamide-resistant MR49F and MR42D cells following 72 h of HIBCH knockdown. **d** mRNA expression of genes encoding the succinyl-CoA generating enzymes within LNCaP cells following 24, 72 and 96 h of siCTR or siHIBCH transfection. **e** mRNA expression of *HIBCH*, *SDHA* and *SUCLG2* following 96 h of induction of shCTR or shHIBCH in LNCaP cells. **f** Metabolomic quantification (LCMS) of tricarboxylic-acid cycle intermediates following 144 h of induction of shCTR or shHIBCH in LNCaP cells. **g** *HIBCH* and (**h**) *MCCC2* mRNA expression following 48, 72 and 96 h of siCTR, siHIBCH or siMCCC2 transfection in LNCaP cells. **i** LNCaP mRNA expression of *ACCa* (*ACACA*), *ACAT1*, *GOT2*, CPT1A, *HMGCR* and *HMGCS* by qRT-PCR following 96 h of siCTR or siHIBCH transfection. All error bars represent SEM. Significance was determined by one-way ANOVA comparing each observation to the vehicle control (siCTR or shNT). ns not significant, *p < 0.05, **p < 0.01, ***p < 0.001, ****p < 0.0001.

To investigate the effects of inhibiting valine catabolism in PCa (Fig. 4a), cell growth was continuously measured following an optimised siRNA-based HIBCH knockdown protocol (Supplementary Fig. 3). LNCaP and PC3 cell confluency and morphology were both significantly impaired at 96 h (Fig. 4b and Supplementary Fig. 4). Cell proliferation was also not reduced in benign BPH-1 cells, suggesting selectivity in malignant but not benign prostate cells. We further confirmed that growth reduction was also associated with reduced levels of intracellular ATP (Supplementary Fig. 5). We also investigated HIBCH silencing in a range of more recently established and clinically representative models of castrate and enzalutamide resistance [21]. Cells which possessed castrate-resistant (V16D), or enzalutamide-resistant (MR49F) phenotypes retained their sensitivity to HIBCH knockdown to an equal or similar degree to androgen-sensitive LNCaP cells co-treated with 10 µM enzalutamide (Fig. 4C). Conversely, we found that PCa cells with neuroendocrine properties (MR42D) [22] were insensitive to HIBCH knockdown, consistent with our investigations in patient metastatic tumours (Fig. 4C).

Expression of genes responsible for succinyl-CoA synthesis (*PCCA, PCCB, MCEE, MUT*) were measured by quantitative reverse transcription PCR (qRT-PCR) at multiple time points following HIBCH knockdown. Expression of these genes were reduced at 48 h with more substantial reductions 96 h post-transfection. This included a significant reduction (~50%) in *MCEE* gene expression (*p* = 0.0004) and a non-significant reduction (~20%) in *PCCA* and *PCCB* expression compared to siCTR (Fig. 4d). HIBCH protein expression was also measured at 72 h to validate knockdown (KD) which confirmed reduced expression in the BPH-1 (69% KD) LNCaP (66% KD) and PC3 (76% KD) prostate cell lines (Supplementary Fig. 6). To investigate the effects of HIBCH suppression on succinate metabolism, doxycycline-inducible short hairpin RNA (shRNA) targeting HIBCH (shHIBCH), and non-targeting control (shNT) LNCaP cells were generated. This model revealed reductions in gene expression of *SDHA* (~50%, succinate oxidation) and *SUCLG2* (~40%, succinate generation) following 72 h of induction (Fig. 4e). We also performed 96 h of HIBCH suppression in LNCaP cells by siRNA, which resulted in less substantial but statistically significant reductions to the expression of both the catalytic (*SDHA*: *p* = 0.010, *SDHB*: *p* = 0.004) and membrane bound (*SDHC*: *p* = 0.032, *SDHD*: *p* = 0.003) subunits of SDH, suggesting a sustained reduction in succinate metabolism (Supplementary Fig. 7).

To functionally confirm these results, a metabolomic analysis was conducted in LNCaP cells as a proof-of-principle experiment to demonstrate the effect of long-term (144 h) shRNA-mediated inhibition of HIBCH. Substantial reductions to succinate (−39%), succinyl-CoA (−32%), fumarate (−46%), malate (−21%), cis-aconitate (−40%) and citrate (−78%) were observed within the shHIBCH samples (Fig. 4f*,* Supplementary Table 4). In contrast, minimal fluctuations to acetyl-CoA (−6.5%), isocitrate (−9.4%), and alpha-ketoglutarate (+5.4%) were detected. Taken together, our results highlight a role for HIBCH in the maintenance of succinate via succinyl-CoA. In addition to valine catabolism, the leucine-specific enzyme methylcrotonyl-CoA carboxylase subunit 2 (MCCC2) was also investigated given its previously published role in PCa, and to ensure it was not enhanced in compensation to reduced HIBCH levels [23]. Following 72 and 96 h of HIBCH knockdown in LNCaP cells, *MCCC2* gene expression was significantly reduced (50%), suggesting a delayed reduction to leucine catabolic activity in response to suppressed valine catabolism (Fig. 4g). Similarly, inhibition of *MCCC2* resulted in significant reductions to *HIBCH* gene expression by 48 h post-knockdown (Fig. 4h), suggesting that valine and leucine catabolism are indirectly linked. An analysis of broader central metabolic genes also showed a statistically significant reduction in the expression of *ACCα (ACACA)* (*p* = 0.0031) and *GOT2* (*p* = 0.0298), and a non-significant reduction in *CPT1A* (*p* = 0.082) (Fig. 4i) Inversely, a significant increase in the expression of *ACAT1* (*p* = 0.0005) was observed, suggesting that compensation of isoleucine catabolism may be invoked in response to reduced valine catabolic activity. Finally, non-significant increases in the expression of *HMGCR* (*p* = 0.1862) and *HMGCS* (*p* = 0.0897) were observed, indicating that cholesterol synthesis may be enhanced following HIBCH knockdown (Fig. 4i)*.*

### HIBCH knockdown inhibits mitochondrial respiration and glycolysis in PCa

**Fig. 5:**
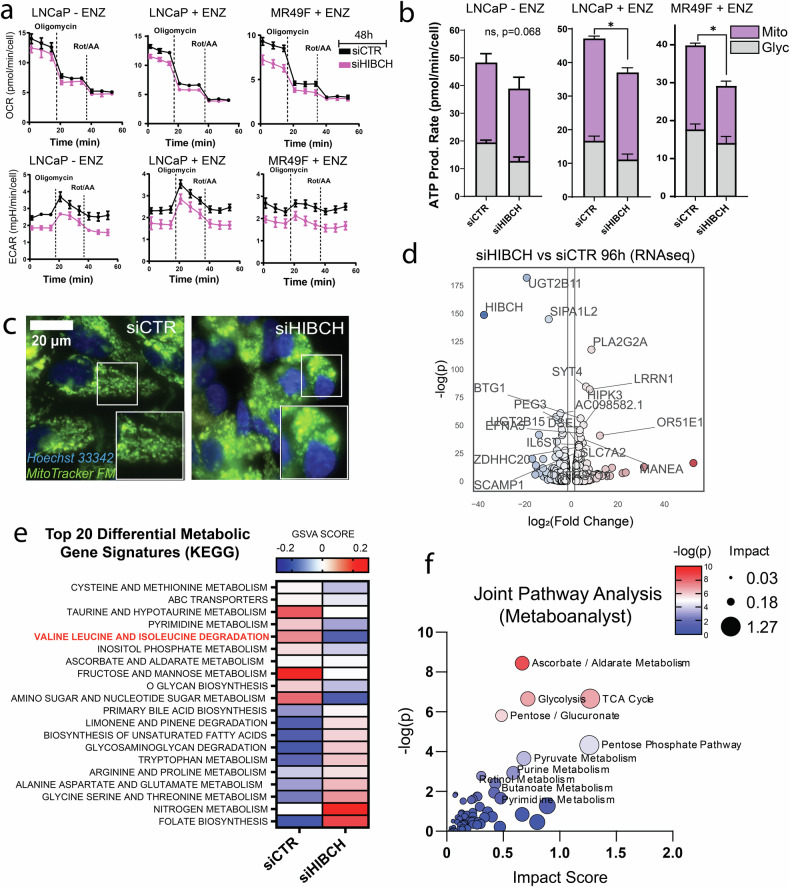
Targeting HIBCH in PCa disrupts oxidative respiration, glycolysis and central metabolic pathways. **a** Oxygen consumption rate (OCR) and extracellular acidification rate (ECAR) following 96 h of siCTR or siHIBCH transfection in LNCaP and MR49F cells measured by Seahorse Extracellular Flux Analysis. **b** Glycolytic vs. mitochondrial ATP production rate calculated from Seahorse Extracellular Flux assay shown in Fig. 5a. **c** Mitochondrial morphology (MitoTracker Green FM) of MR49F cells following 72 h of siCTR or siHIBCH transfection. **d** Volcano plot showing most differentially expressed genes following HIBCH knockdown. **e** Heatmap of the top 20 differential gene signature scores of LNCaP cells following 96 h of siCTR or siHIBCH transfection measured by RNAseq. **f** Joint pathway analysis of both metabolomic and RNAseq data ranked by -log(p) value and impact scores. Error bars represent SEM. Significance was determined by One-Way ANOVA comparing each observation to the vehicle control (siCTR or shCTR). ns – not significant, *p < 0.05.

To functionally measure the metabolic response of PCa cells to HIBCH knockdown, real-time metabolic flux analysis was performed. From our previous analysis of LNCaP PCa cells, it was uncovered that valine catabolism is enhanced in response to enzalutamide. We, therefore, sought to compare the glycolytic and mitochondrial response of enzalutamide sensitive (LNCaP) and resistant (MR49F) cells in response to 48 h of HIBCH knockdown to identify acute changes in cellular respiration. LNCaP cells were exposed to either vehicle (EtOH) or 10 µM enzalutamide (ENZ) for 48 h while MR49F cells were maintained in continuous ENZ to prevent therapeutic re-sensitisation. Following treatment and siRNA transfection, glycolytic lactate export was calculated as a function of extracellular acidification rate (ECAR) while oxidative respiration was indicated by oxygen consumption rate (OCR). Basal OCR, ECAR and ATP production rate was reduced in both cell lines following HIBCH knockdown, however this response was less pronounced in LNCaP cells not exposed to ENZ (Fig. 5a, b). Reductions in oxidative respiration were more prominent in MR49F cells, supporting the concept that enzalutamide resistance may lead to a heightened energetic dependency on HIBCH. Live cell fluorescent microscopy of MR49F mitochondria also revealed dramatic changes to mitochondrial morphology, including increased fragmentation of the mitochondrial tubular network (Fig. 5c). To investigate the global transcriptomic consequences induced by HIBCH knockdown, RNA sequencing was performed on mRNA isolated from LNCaP PCa cells following 96 h of siRNA HIBCH knockdown. Analysis revealed 497 differentially expressed genes (269 increased, 228 decreased) in response to knockdown. The top 10 differentially expressed genes included decreased *HIBCH, UGT2B11, UGT2B28* and *SIPA1L2*, and increased *OR51E1, PLA2G2A*, *LRRN1*, *SYT4*, and *HIPK3* (Table 1 and Fig. 5d*).* To investigate changes to differential metabolic pathway enrichment, gene set variation scoring (GSVA) was performed. This revealed downregulation of pathway scores for BCAA degradation, cysteine and methionine metabolism and expression of the ABC transporters, while nitrogen metabolism, folate biosynthesis and unsaturated fatty acid synthesis were inversely upregulated (Fig. 5e and Supplementary Table 5). To improve confidence in the identification of dysregulated metabolic pathways, an integrated analysis was performed with MetaboAnalyst 5.0, to calculate an impact score derived from the previously acquired metabolomics in combination with the RNAseq (Fig. 5f and Supplementary Table 6). This analysis revealed that the top 5 most impacted pathways were ascorbate/aldarate metabolism, TCA cycle, pentose phosphate pathway, glycolysis and pyruvate metabolism.

**Table 1:** Top 10 differentially expressed genes (up and down) following HIBCH knockdown in LNCaP PCa cells.

Gene (up)	Log (FC)	Adj. *P* val	Ensembl99 ID	Gene (Down)	Log (FC)	Adj. *P* val	Ensembl99 ID
OR51E1	12.29	2.09E-18	ENSG00000180785	HIBCH	−37.48	3.11E-65	ENSG00000198130
PLA2G2A	8.63	9.28E-52	ENSG00000188257	UGT2B11	−19.11	1.15E-79	ENSG00000213759
LRRN1	7.89	2.50E-36	ENSG00000175928	UGT2B28	−13.87	1.01E-18	ENSG00000135226
LCN2	7.45	1.95E-05	ENSG00000148346	SIPA1L2	−9.69	1.41E-63	ENSG00000116991
SYT4	6.27	2.04E-37	ENSG00000132872	ITGA1	−6.78	4.41E-10	ENSG00000213949
CRYAB	5.95	2.25E-04	ENSG00000109846	F5	−6.46	1.23E-25	ENSG00000198734
HIPK3	5.25	1.39E-22	ENSG00000110422	AGR2	−6.21	1.22E-12	ENSG00000106541
PXYLP1	5.13	7.83E-05	ENSG00000155893	FAR2P3	−6.20	8.07E-04	ENSG00000240253
PRSS1	5.10	2.78E-03	ENSG00000204983	ZNF812P	−6.15	4.36E-16	ENSG00000224689
SLC7A2	4.87	1.77E-15	ENSG00000003989	ODAM	−5.87	5.88E-04	ENSG00000109205

## Discussion

Metabolic plasticity underpins the evasive nature of cancer. We have uncovered novel insights into the role of valine catabolism in maintaining SLRF in advanced PCa. This work links, for the first time, the previously reported metabolic derangements in PCa, BCAA degradation, lipid metabolism and oxidative phosphorylation. While the BCAAs have been previously reported to be critical regulators of lipid biology within both adipose and hepatic tissues [17, 24, 25], our investigation has shed light on their importance in malignant prostate cells. Our data demonstrates that extracellular BCAAs are essential to maintain intracellular lipid reserves in multiple models of advanced and castrate-resistant PCa (LNCaP, C4-2B and PC3). This finding was further supported in our BCAA catabolic inhibitory models, which demonstrated that knockdown of BCAT1 could successfully reduce lipid content in LNCaP and PC3 cells, while knockdown of BCAT2 results in an increase to lipid content in PCa cells, but not in benign BPH-1 cells. Gene expression analysis of *BCAT1* and *BCAT2* following their knockdown also confirmed a compensatory transcriptional relationship, highlighting a dynamic cross-regulatory mechanism. We identified an important specific role for valine in co-regulating fatty acid uptake in androgen-responsive PCa. In the absence of extracellular valine, LNCaP cells increase long-chain fatty acid uptake, a result supported by other studies that link valine catabolism and fatty acid uptake through a regulatory feedback loop between HIBCH and pyruvate dehydrogenase kinase 4 (PDK4) [25]. This study also found that these changes were exacerbated in the absence of exogenous fatty acids, which may be explained by our discovery of increased fatty acid uptake in response to valine deprivation. This response was unique to PCa cells but absent in non-malignant BPH-1 cells, which instead demonstrated a unique response to leucine deprivation. Together, our investigations suggest a possible switch in metabolic dependency in PCa cells, including a modified reliance from BCAT2 to BCAT1 and from extracellular leucine to valine. Future studies would benefit from investigations into the dual knockdown of BCAT1 and 2 and in the presence and absence of extracellular lipids to resolve the dynamic relationship between BCAT isoforms.

Recently, enhanced succinate oxidation by SDH has been identified as an emergent hallmark of PCa metabolism [10–12]. Increased reliance on succinate in response to reduced Complex I (CI) activity is also not unique to cancer, as cell-permeable succinate has been shown to overcome CI dysfunction in models of mitochondrial disease [26]. Direct inhibition of SDH activity has been explored as a therapeutic strategy within multiple cancers due to its critical role in oxidative phosphorylation, however, an inherent flaw with this approach is the resultant accumulation of intracellular succinate. Increased cytoplasmic succinate is well known to stabilise hypoxia-inducible factors (HIFs), which exacerbate disease progression in multiple malignancies [27, 28]. As a result, cells which are deficient in SDH activity are also subject to enhanced pseudohypoxia, epithelial to mesenchymal transition (EMT) and metastatic potential [29, 30]. Our unique approach has aimed to resolve this problem, by instead inhibiting the production of succinate precursors via valine catabolism. Our analyses of publicly available data confirmed transcriptomic enrichment of genes which facilitate the entry of valine into the TCA cycle (MitoS gene signature) in patient PCa tumours, which negatively correlates to patient survival.

Inhibition of valine catabolism via the suppression of HIBCH, a key enzyme facilitating the conversion of 3HIB-CoA to 3HIB, results in metabolic catastrophe for PCa cells, including reduced intracellular succinate among other TCA metabolites, reduced oxidative and glycolytic function, induction of mitochondrial fragmentation and reduced cell growth and viability. Despite our identification of AR^high^ tumours experiencing enhanced MitoS scores, patients with metastatic double-negative tumours also possessed a similar level of HIBCH expression, possibly explaining the heightened sensitivity of AR^null^ PC3 cells to HIBCH inhibition_._ What is clearer from our analysis however is that the presence of neuroendocrine features is likely a more definitive indicator of poor response to HIBCH inhibition. This is supported by our investigation into MR42D cells which showed no response. Our findings are also supported by Shan et al. [18], who demonstrated that HIBCH inhibition in colorectal cancer cells reduced cell proliferation and TCA metabolite levels [18]. We also uncovered a significant reduction of intracellular citrate with minimal changes to acetyl-CoA. These results are consistent with other studies which have knocked down HIBCH and found reduced levels of fumarate, malate and citrate with no changes to α-ketoglutarate [25]. The unexpected lack of change to the acetyl-CoA levels is another interesting question for future investigations quantifying cytosolic and mitochondrial acetyl-CoA pools and their directly connected pathways. Furthermore, in response to HIBCH inhibition a time-delayed reduction to *MCCC2* gene expression was also observed, a key enzyme in the leucine catabolic pathway. MCCC2 has been previously highlighted as a therapeutic target in PCa [23] and was measured to validate that leucine catabolism was not enhanced in response to HIBCH inhibition. Our findings suggest that not only do MCCC2 and HIBCH fail to share a compensatory relationship but are mutually dependent on the activity of the other. Our exploration into the global multi-omic changes occurring because of HIBCH inhibition also highlighted several broader metabolic consequences including reduced gene expression of *ACCα* and *GOT2*, and dysregulation of the ascorbate, cysteine/methionine, nitrogen, and folate metabolic pathways.

Our study has not examined alternative sources of succinyl-CoA, including the catabolism of isoleucine, methionine, and threonine, however, amongst these, valine remains the most abundant and readily available amino acid in PCa patient serum (Val: 222.8 µM, Ile: 56.3 µM, Met: 16.9 µM, Thr: 102.1 µM) [31]. Our findings also highlight the significance of the specific roles of each BCAA in cellular metabolism and justify further investigation of their unique functions and regulation. This will be essential in further understanding the specific energetic demands which are dynamically adapted throughout the PCa disease spectrum and will assist in the generation and deployment of novel metabolically targeted strategies. Future studies would also benefit from investigation of these findings in models more representative of normal prostatic biology. However, given the lack of available primary epithelial cell lines grown in comparable culture conditions, BPH-1 cells were deemed suitable for this study. More broadly, ontological pathway databases would likely benefit from separating the leucine, isoleucine, and valine catabolic entries to prevent statistical muting introduced from signature-based analyses, which often group BCAA degradation as a singular process. Succinate has been identified as having many pro-oncogenic roles, including HIF1α stabilisation [27, 28], alteration of the tumour microenvironment [32], promotion of metastatic potential via enhanced EMT [29], suppression of anti-tumour immune responses [33, 34] and the activation of pro-inflammatory signalling pathways [35]. Future directions should investigate the effects of HIBCH inhibition on these processes. In summary, our study introduces a novel therapeutic approach which exploits metabolic reliance on succinate in PCa. This multifaceted investigation reveals the intricate connections between BCAAs, lipid metabolism, and succinate-linked respiration in the context of PCa. The clinical implications of our findings highlight the potential of HIBCH inhibition as a targeted therapeutic strategy for PCa, warranting further exploration and validation.

## Methods and materials

### Cell culture

Benign (BPH-1) and malignant human prostate cell lines (LNCaP, C4-2B, PC3) were obtained from the ATCC and maintained in RPMI 1640 (Gibco) supplemented with 5% foetal bovine serum (FBS) at 37 °C / 5% CO_2_. Permission to use castrate and enzalutamide-resistant human PCa cell lines V16D, MR49F and MR42D was granted by A. Zoubeidi from The University of British Columbia (UBC). Cells were continuously maintained in RPMI 1640 with 10% FBS at 37 °C / 5% CO_2_. MR49F and MR42D cells were additionally co-cultured in 10 µM enzalutamide (MedChemExpress) to prevent therapeutic re-sensitisation. All cells were cultured between passage number 20–40 and their medium replenished every 48–72 h. Cells were routinely screened for mycoplasma using the Translational Research Institute’s routine mycoplasma testing service and originally STR profiled by the QUT Genomics Research Centre.

### Amino acid depletion assays

Following 72 h of standard culture in poly-L-ornithine coated 96-well optical plates (CellVis), culture medium was washed twice and refreshed with BCAA-depleted medium (BDM). BDM was generated though supplementation of glucuncoverose and amino acids (except BCAAs) to RPMI 1640 without l-Glutamine, Amino acids, Glucose or Phenol Red (MyBioSource). BDM was supplemented with 10% dialysed FBS to prevent the addition of amino acids from serum. Exogenous supplementation of leucine (100 µM), isoleucine (55 µM) and valine (220 µM) were then performed to restore physiologically representative BCAA serum concentrations. The formulation of BDM and specific catalogue numbers have been included in Supplementary Table 1.

### Small interfering RNA knockdown

siRNA silencing was accomplished using forward transfection of pre-designed MISSION siRNAs from Sigma-Aldrich. A 10 nM final concentration of siBCAT1 (SASI_Hs01_00066058), siBCAT2 (SASI_Hs02_00331330), siHIBCH (SASI_Hs01_00064760) or siMCCC2 (SASI_Hs01_00039625) was transfected into cells with RNAiMAX (ThermoFisher Scientific) diluted 1:25 in serum-free RPMI 1640. Following 5 h of transfection, standard serum conditions were restored by the addition of FBS (5%). A universal negative control (Sigma Aldrich) with no known human homologies were included in all experiments to control for non-specific toxicity.

### Inducible short hairpin RNA knockdown

Glycerol *Escherichia coli* stocks containing SMARTvector inducible lentiviral shRNA (mCMV, tGFP) were obtained from Horizon Discovery. Purified plasmid DNA was generated from overnight LB cultures with the QIAprep Spin Miniprep Kit (Qiagen) and quantified. Viral supernatants were generated by transfection of HEK293T (ATCC) cells with 0.2 µg pCMV delta R8.2 (lentiviral packaging plasmid), 1.8 µg pCMV-VSV-G (lentiviral envelope plasmid) and 2 µg purified shRNA DNA with 12 µg (3:1) of X-tremeGENE transfection reagent (Sigma Aldrich). Following 72 h of transfection, LNCaP cells were transduced with fresh viral supernatant for 6 h and the cultures expanded. Target-specific regions of shHIBCH1 and shHIBCH2 were ***GAATAATGCTGTTGGTTCT*** and ***ACTCTTAATATGATTCGGC****,* respectively.

### Quantitative reverse transcription PCR

Following cell siRNA transfection or shRNA induction, total RNA was isolated using the Total RNA Purification Plus Kit (Norgen) according to the manufacturer’s protocol. RNA concentration was quantified, and cDNA generated from 1 µg of RNA using the SensiFAST cDNA synthesis kit (Bioline). cDNA was then diluted (1:5) with RNAase/DNAase-free water and qRT-PCR performed using SYBR-Green Master Mix (ThermoFisher Scientific) and primers listed in Supplementary Table 2. qPCR was monitored on the ViiA-7 Real-Time PCR system (Applied Biosystems, Waltham, USA). Relative mRNA expression was then calculated using the ∆∆CT method normalising gene expression to the RPL32 house-keeping gene.

### SDS-PAGE and western blotting

Cells were scraped and lysed with 100 µL of freshly prepared RIPA buffer. Samples were centrifuged at 14,000 RCF for 15 minutes before collection of the supernatant and removal of cellular debris. Protein concentration was measured by BCA assay and 50 µg of lysate prepared using Bolt™ reducing and loading buffers (ThermoFisher Scientific). Samples were run on a 4-12% Bis-Tris Mini Protein Gel (ThermoFisher Scientific) at 120 V/60 mins in MOPS buffer. Nitrocellulose membrane transfer was conducted in transfer buffer at 10 V for 1 hour, before blocking in Odyssey TBS Blocking Buffer (LI-COR) for 1 hour at room temperature. HIBCH (HPA036540, Sigma Aldrich) and y-Tubulin (AB11316, Abcam) antibodies were diluted 1:1000 and incubated with the membrane overnight at 4 °C. The membrane was subsequently incubated with IRDye® secondary antibodies (LICOR) at 1:25000 for 1 hour at room temperature protected from light. Imaging was performed using the Odyssey infrared imaging system (LI-COR, Lincoln, USA).

### Intracellular lipid content quantification

Following 24 h of culture in BDM, media was removed, and the optical plate was washed twice with 200 µL PBS. Cells were fixed with 4% paraformaldehyde (PFA) at room temperature for 30 minutes, rinsed with PBS, then intracellular lipids co-stained with 0.1 µg/mL Nile Red (Invitrogen) and 1 µg/mL 4,6-diamidino-2-phenylindole (DAPI, Invitrogen) in PBS for 1 hour at 4 °C. Automated fluorescent microscopy was accomplished using the InCell Analyzer 6500HS (Cytiva, Amersham, United Kingdom). Image segmentation and quantitation of cellular parameters from generated images were derived using custom pipelines in CellProfiler 4 [36]. Representative images (60X) where processed with ImageJ and identical thresholding settings were applied across all images where comparisons have been made [37].

### Live cell C16:0-BODIPY uptake assay

Following 24 h of culture in BDM, media was removed, and the optical plate was washed twice with PBS. Cells were then treated with 5 µM C16:0 BODIPY (C16) (Invitrogen), 1 µg/ml Hoechst 33342 (ThermoFisher Scientific) and 0.2% bovine serum albumin (BSA) (Sigma Aldrich) in serum-free BDM for 30 min at 37 °C to allow cellular uptake of C16. Cells were washed twice with serum-free BDM before imaging. Images were captured using the InCell Analyzer 6500HS (Cytiva, Amersham, United Kingdom) and image analysis was performed as described above.

### Live/dead staining and analysis

Cells were incubated with 1 µg/ml Hoechst 33342 (ThermoFisher Scientific) and 1 μg/mL Propidium Iodide (Invitrogen) in serum-free RPMI 1640 for 30 minutes at 37 °C / 5% CO_2_. Following incubation, cells were imaged using the InCell Analyzer 6500HS (Cytiva, Amersham, United Kingdom). Cell death was quantified as a percentage of propidium iodide-positive cells divided by the total number of cells identified by Hoechst staining using CellProfiler 4 [36].

### Seahorse extracellular flux assay

Quantification of glycolytic and mitochondrial ATP production was performed using the Seahorse XFe Real-Time ATP Rate Assay Kit (Agilent). Prior to cell adhesion, Seahorse 96XFe tissue culture plates were coated with 0.01% poly-l-ornithine solution (Sigma-Aldrich) and incubated at 37 °C. Cells were seeded at a density of 10,000 cells per well in standard phenol red-free RPMI 1640 (ThermoFisher Scientific) with 5% FBS. Cells were subject to 48 h of siRNA mediated HIBCH knockdown as described above. At endpoint, the media was replaced with Seahorse Base Medium (Agilent), supplemented with 2 mM L-glutamine (Sigma Aldrich), 10 mM glucose (ThermoFisher Scientific) and 1 mM sodium pyruvate (ThermoFisher Scientific) adjusted to pH 7.4. Oligomycin (15 µM) and a combined injection of 10 µM antimycin A + 10 µM rotenone were added to the appropriate Seahorse XFe injection ports. The Seahorse XFe96 analyser (Agilent Technologies, Santa Clara, USA) was programmed to measure both oxygen consumption rate (OCR) and extracellular consumption rate (ECAR) over the course of 60 minutes. Nine measurements were taken at 7-minute intervals with oligomycin injected between measurements 3 and 4 and rotenone + antimycin A injected between measurements 6 and 7. Extracellular flux data was analysed using Wave desktop software (Agilent) and normalised to the number of cells in each well quantified with a Hoechst nuclear stain.

### LCMS metabolite quantification

shHIBCH and shNT LNCaP cells were induced or not induced with 0.25 µg/mL doxycycline for 144 h. Cells were rapidly washed with 5 mL isotonic wash solution (5% d-mannitol) on ice to remove residual media and exogenous metabolites without disturbing metabolite integrity as previously described [38]. Instantly, 1 mL of ice-cold extraction buffer (50% methanol + 250 nM azidothymidine internal standard) was added before cells were scraped and transferred into to 15 mL conical tubes. This step was repeated, and the two fractions were combined. Samples were freeze-thawed (−80 °C) then sonicated on ice for 10 minutes. Metabolites were purified from the organic fraction of a 25:24:1 phenol:chloroform:isoamyl solution and centrifuged at 16,000 RCF for 5 minutes at 4 °C. The supernatant was removed without disturbing the interface layer, frozen and sent to the Metabolomics Australia Queensland Facility for further analysis. Samples were freeze-dried and resuspended in 100 µL 2% acetonitrile (ACN) before being injected in two dilutions to account for natural variability in metabolite levels. Samples were analysed using the Shimadzu 8060 LC MS-MS instrument (Shimadzu, Kyoto, Japan) and Gemini NX-C18 3 µm x 150 mm x 2 mm column (Phenomenex, Torrance, USA). Data analysis was subsequently performed using Shimadzu LabSolutions Insight software.

### RNA sequencing

Total cellular RNA from LNCaP cells following 96 h of HIBCH knockdown was extracted using the Norgen RNA Purification PLUS kit (Norgen) according to the manufacturer’s instructions. RNA quality and quantity were determined on an Agilent 2100 Bioanalyzer (Agilent Technologies, Santa Clara, USA) and Qubit® 2.0 Fluorometer (ThermoFisher Scientific, Waltham, USA). Library preparation and sequencing was performed at the QUT Central Analytical Research Facility (CARF) using the Illumina TruSeq Stranded mRNA Sample Prep Kit (strand-specific, polyA enriched, Illumina, San Diego, USA) with an input of 1 µg total RNA (RIN > 9), followed by paired-end sequencing on the Illumina NovaSeq6000. Raw reads were trimmed using TrimGalore, followed by alignment to the human genome (GRCh38 / hg38) using the STAR2 aligner and read quantification with RSEM4 [39, 40]. Differential expression (DE) between siCTR and siHIBCH samples was calculated after sample normalisation using edgeR (no replicates: Fisher Exact Test; replicates: General Linear Model) and is defined by an absolute fold change of ≥1.5 and an FDR corrected *p*-value ≤ 0.05 [41]. Data quality control included running FastQC before and after trimming, checking RNAseq metrics with the PICARD tool kit, and mapping reads against microbial genomes using Kraken [42, 43]. Raw data files (.fastq) and processed gene expression data are accessible through NCBI GEO series accession number GSE253413.

### Statistical and bioinformatic analysis

Statistical analyses were performed using either GraphPad Prism 10 or the SciPy python package [44]. Specific statistical tests employed are described in the legends below each figure. Analysis of publicly available RNAseq datasets were carried out by first reprocessing each study’s raw data obtained via the NCBI Gene Expression Omnibus (GEO) or the European Genome-phenome Archive (EGA) through our in-house RNAseq processing pipeline as detailed above. Hierarchically clustered heatmaps were produced by applying the seaborn (python package) ClusterMap function on z-scored Fragments Per Kilobase of transcript per Million (FPKM) values for each respective gene. Kaplan–Meier survival analyses were performed using Lifelines (python package) on FPKM expression values of patient data grouped by the highest (*n* = 30) and lowest (m = 30) expressing individuals of each respective gene or signature. Gene signature scoring was accomplished using the Gene Set Variation Analysis (GSVA) package in R, incorporating each genes respective transcript per million (TPM) value and respective gene set from the KEGG metabolic gene signature repository [45]. Joint pathway analysis was achieved using MetaboAnalyst 5.0 using differentially expressed fold change values (FC ≥ 1.5, *p* ≤ 0.05) derived from the RNAseq and metabolomic experiments listed above [46].

### Supplementary information

Original Data - Western Blots

Original Data - qRT-PCR

Supplementary Figures 1-7

Supplementary Tables 1-6

## Data Availability

Publicly available datasets analysed for the purpose of this manuscript are available from the European Genome-Phenome Archive under accession number EGAD00001005931 (Schöpf et al. [12]), the NCBI Gene Expression Omnibus (GEO) under accession numbers: GSE143408 (Tousignant et al. [5]) and GSE126078 (Labrecque et al. [19]) and cBioPortal datasets PRAD_SU2C_2019 (Abida et al. [20]) and The Cancer Genome Atlas’ (TCGA) Prostate Adenocarcinoma (TCGA-PRAD) cohort. RNA sequencing data produced in this study has been deposited to NCBI GEO under the accession number: GSE253413.
