# Supplementary material for: Targeting valine catabolism to inhibit metabolic reprogramming in prostate cancer
Source: Cell Death Dis. 2024 Jul 18;15(7):513. doi: 10.1038/s41419-024-06893-2 (PMC11258138; doi:10.1038/s41419-024-06893-2)
Supplement: Supplementary file 1 — Original Data - Western Blots [file 41419_2024_6893_MOESM1_ESM.pdf]

Supplementary Figure 3a

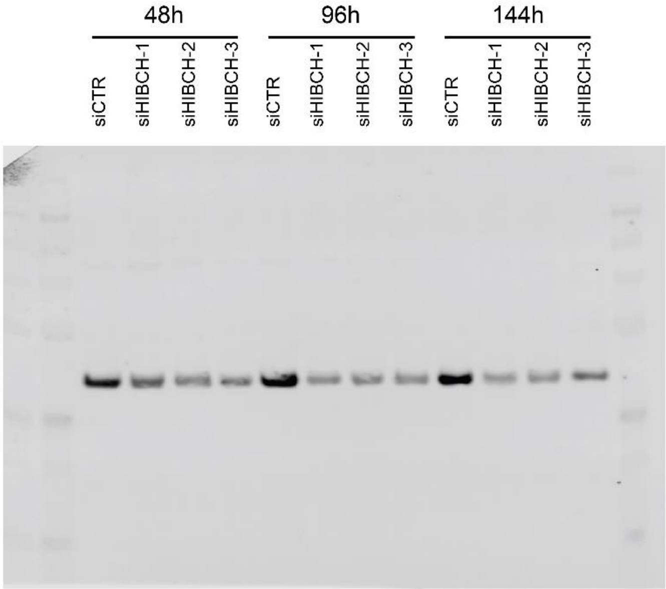

anti-HIBCH Rabbit (HPA036540, Sigma Aldrich)

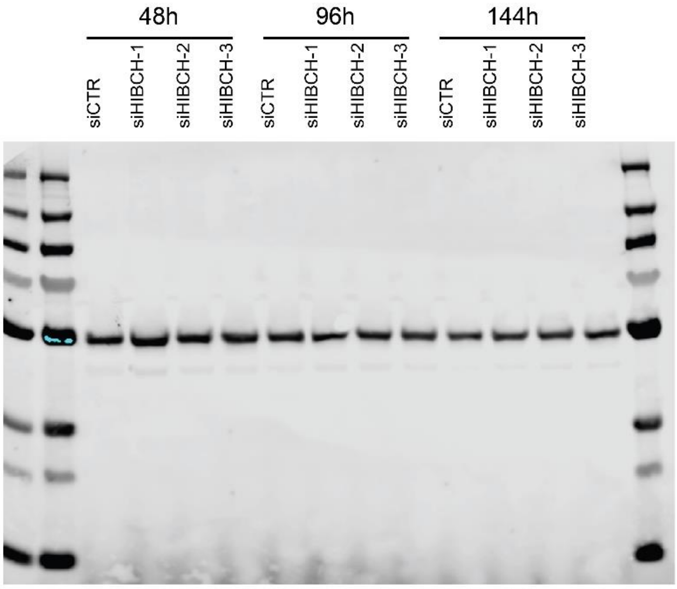

anti-γ-Tubulin Mouse (ab11316, Abcam)

Supplementary Figure 4a

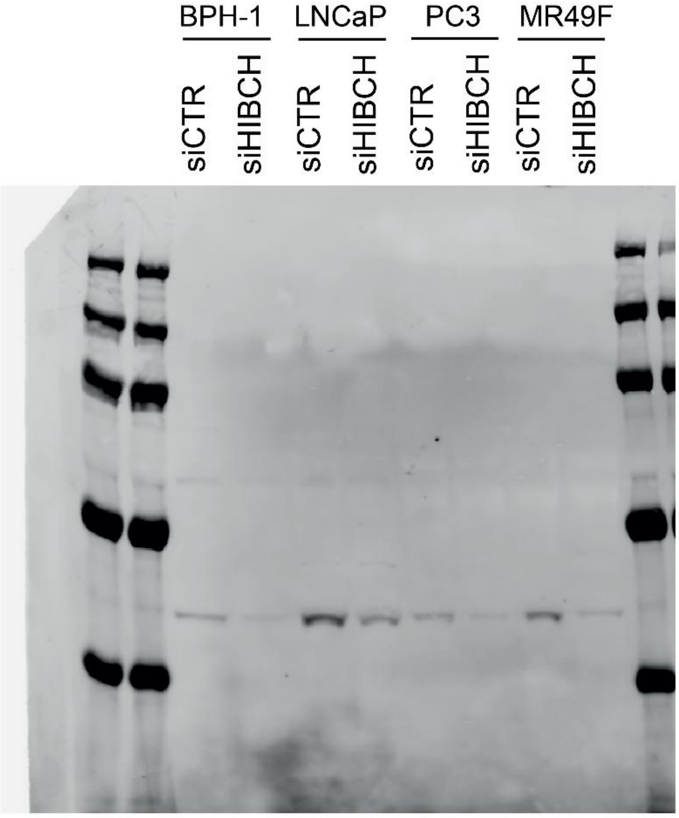

anti-HIBCH Rabbit (HPA036540, Sigma Aldrich)

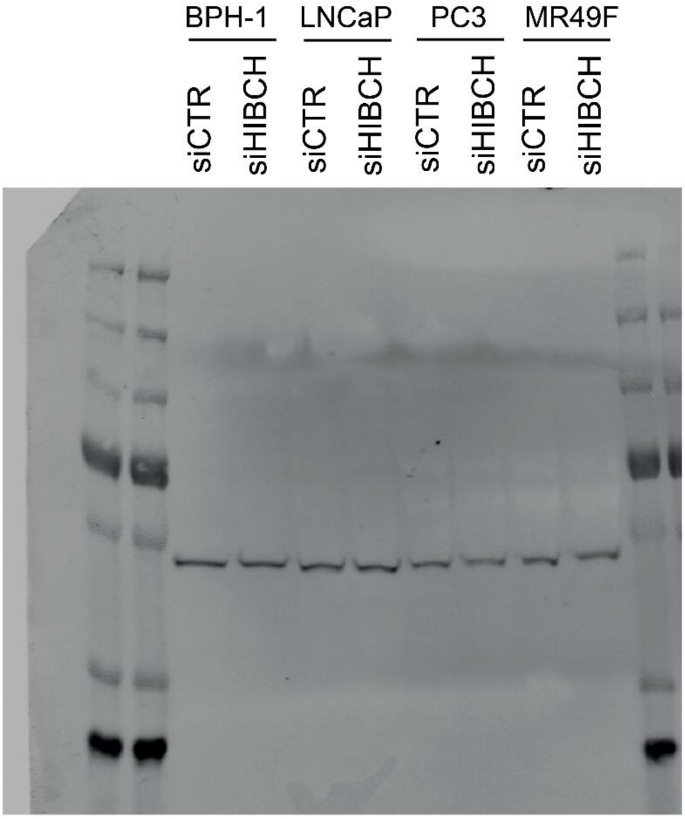

anti-γ-Tubulin Mouse (ab11316, Abcam)
