# Supplementary material for: Targeting valine catabolism to inhibit metabolic reprogramming in prostate cancer
Source: Cell Death Dis. 2024 Jul 18;15(7):513. doi: 10.1038/s41419-024-06893-2 (PMC11258138; doi:10.1038/s41419-024-06893-2)
Supplement: Supplementary file 3 — Supplementary Figures 1-7 [file 41419_2024_6893_MOESM3_ESM.docx]

**Targeting Valine Catabolism to Inhibit Metabolic Reprogramming in Prostate Cancer**

Charles L. Bidgood^1†^, Lisa K. Philp^1^, Anja Rockstroh^1^, Melanie Lehman^1,2^, Colleen C. Nelson^1^, Martin C. Sadowski^3^, and Jennifer H. Gunter^1†^

_† Corresponding Authors_

1. Australian Prostate Cancer Research Centre - Queensland, Queensland University of Technology, Translational Research Institute, Brisbane, QLD 4102, Australia

2. Vancouver Prostate Centre, Department of Urologic Sciences, University of British Columbia, 2660 Oak St, Vancouver, BC V6H 3Z6, Canada.

3. University of Bern, Institute for Tissue Medicine and Pathology, Murtenstrasse 31, CH-3008 Bern, Switzerland

**Supplementary Figures**


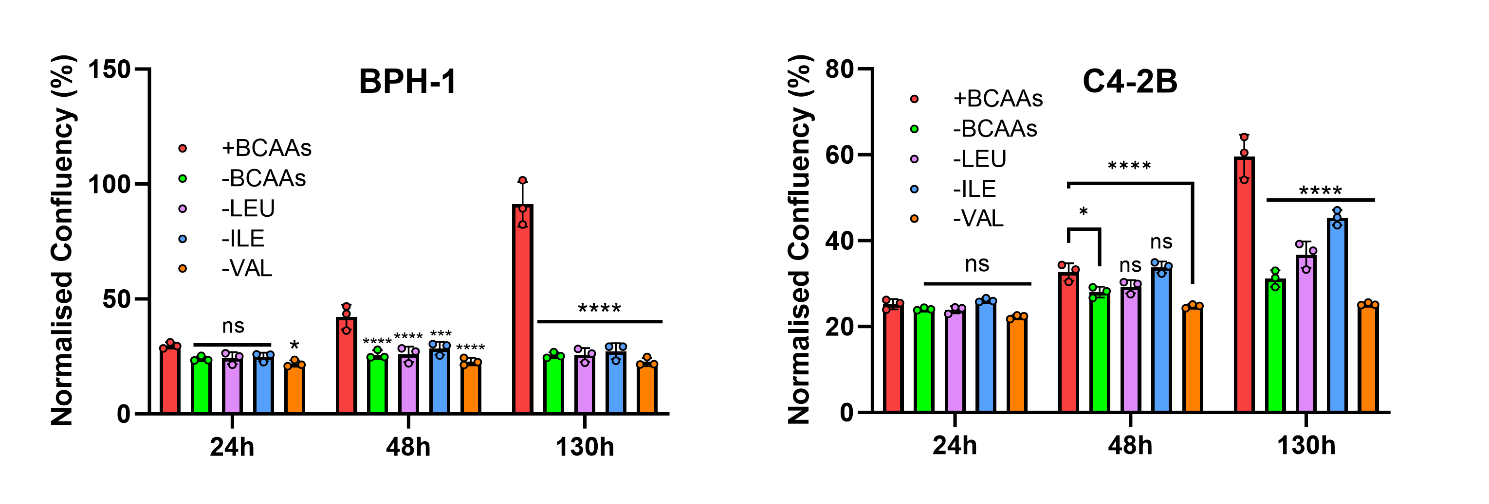


***Supplementary Fig. 1:*** ***Time-Dependent Prostate Cell Growth Following Differential BCAA Starvation.*** *Normalised cell confluency values of non-malignant BPH-1 and malignant C4-2B PCa cells following 24, 48 and 130h of differential branched-chain amino acid (BCAA) depletion (LEU – Leucine, ILE – Isoleucine, VAL – Valine). Statistical analysis performed with two-way ANOVA with Dunnett’s Multiple Comparison Test, ns - not significant, *p<0.05, ***p<0.001 ****p<0.0001.*


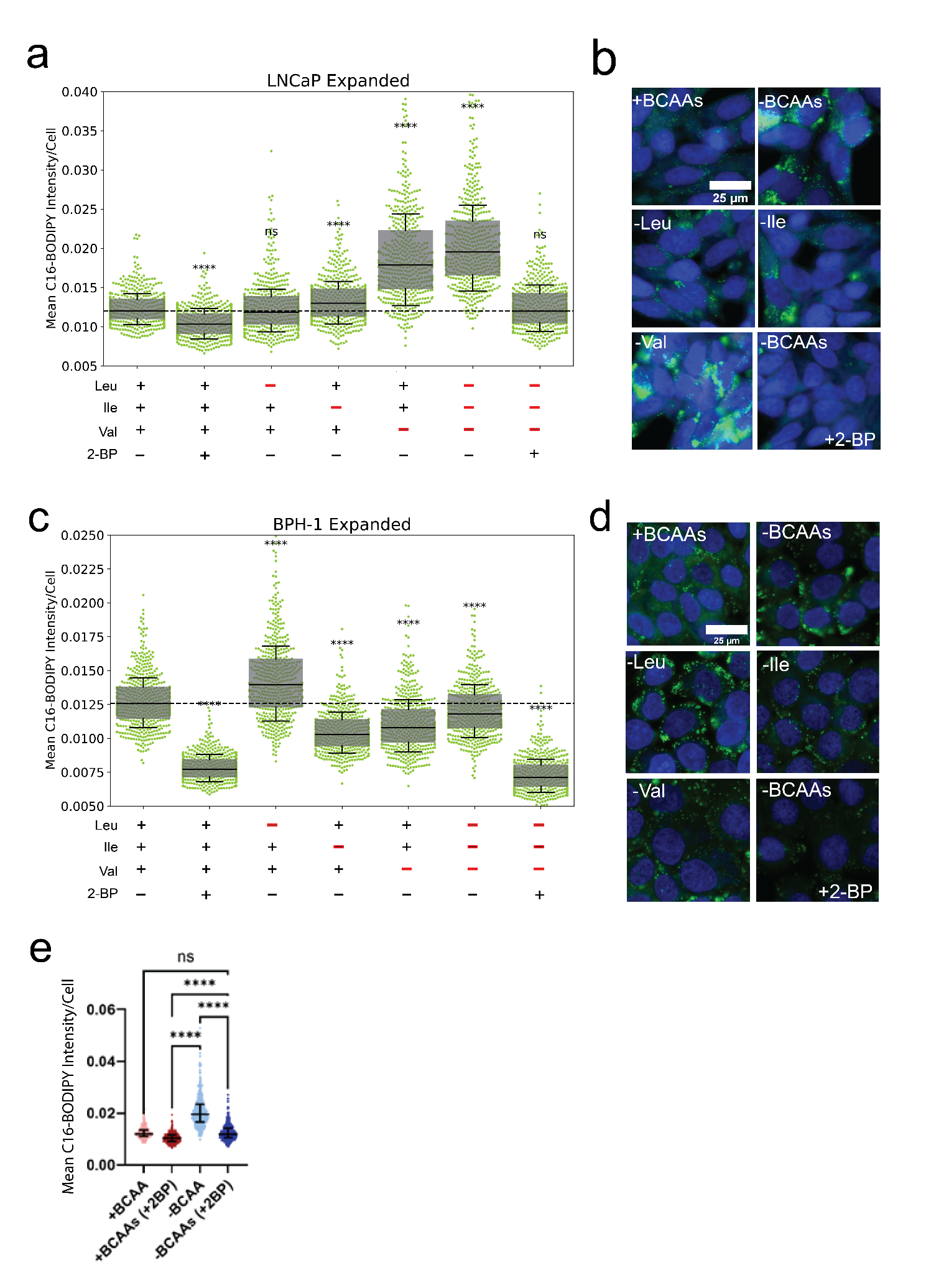


***Supplementary Fig. 2: Differential BCAA Deprivation Triggers C16:0 Fatty Acid Uptake in Prostate Cells****. Quantitative single cell imaging (qSCI) analysis* ***(a,c)*** *and representative live-cell fluorescent images* ***(b,d)*** *of C16-BODIPY uptake following 24h of exogenous BCAA deprivation and/or co-treatment with 2-Bromopalmitate 2-BP in BPH-1 and LNCaP cells.* ***(e)*** *Tukey’s Multiple Comparison Test showing statistical comparisons between +/-BCAAs and +/-2-Bromopalmitate (2BP).*

***
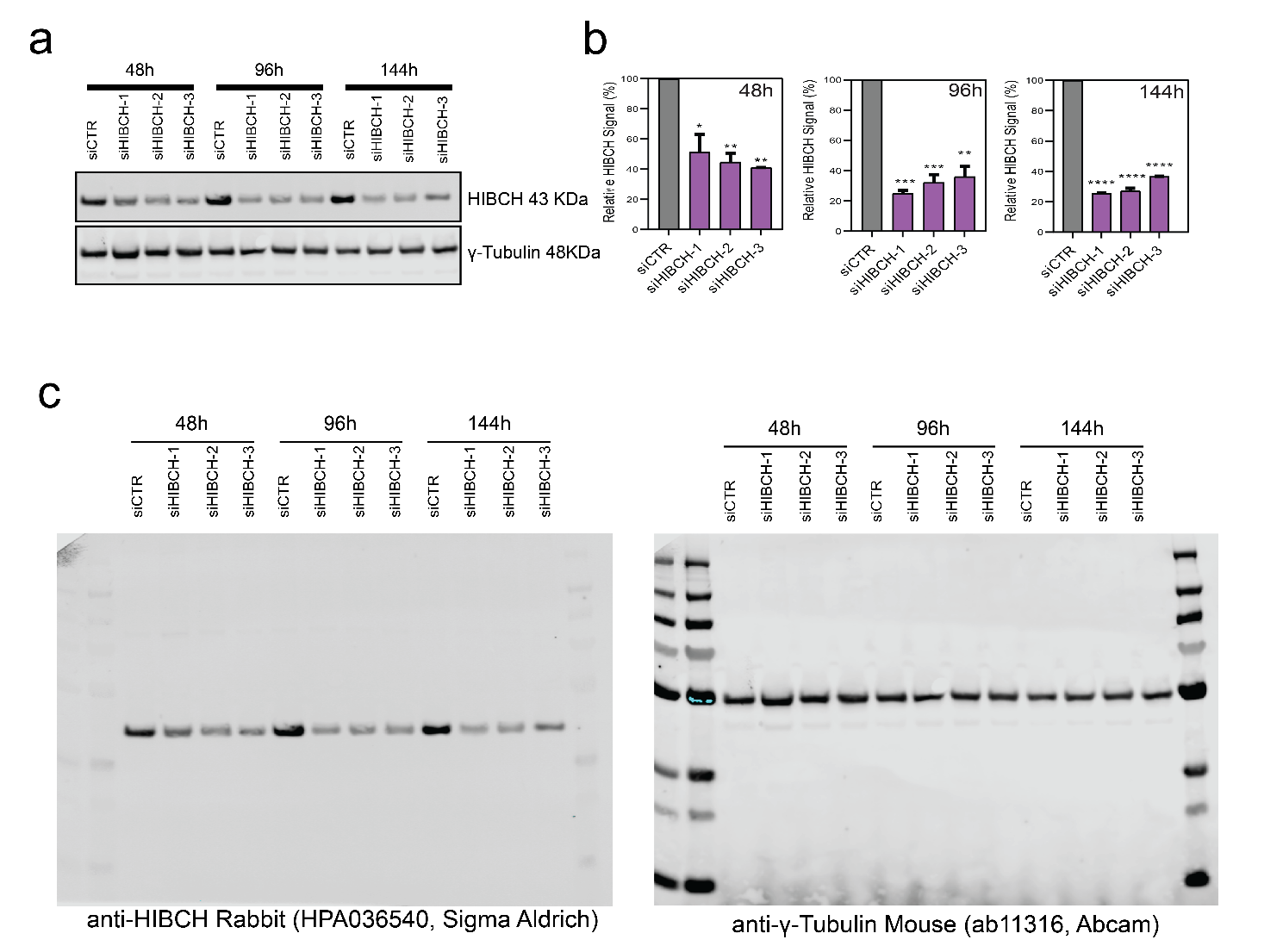
***

***Supplementary Fig. 3: Optimisation and Confirmation of HIBCH knockdown by siRNA Transfection*** *Comparison of three HIBCH siRNAs was performed in LNCaP PCa cells to determine the most effective and stable siRNA sequence. siHIBCH-1 (MISSION siRNA* #*SASI_Hs01_00064760) resulted in the highest knockdown efficiency at 144h in LNCaP cells quantified by western blot (****a****-****b****) and was thus selected for use within the present study. Full western blots with antibody catalogue numbers are included above (****c****).*


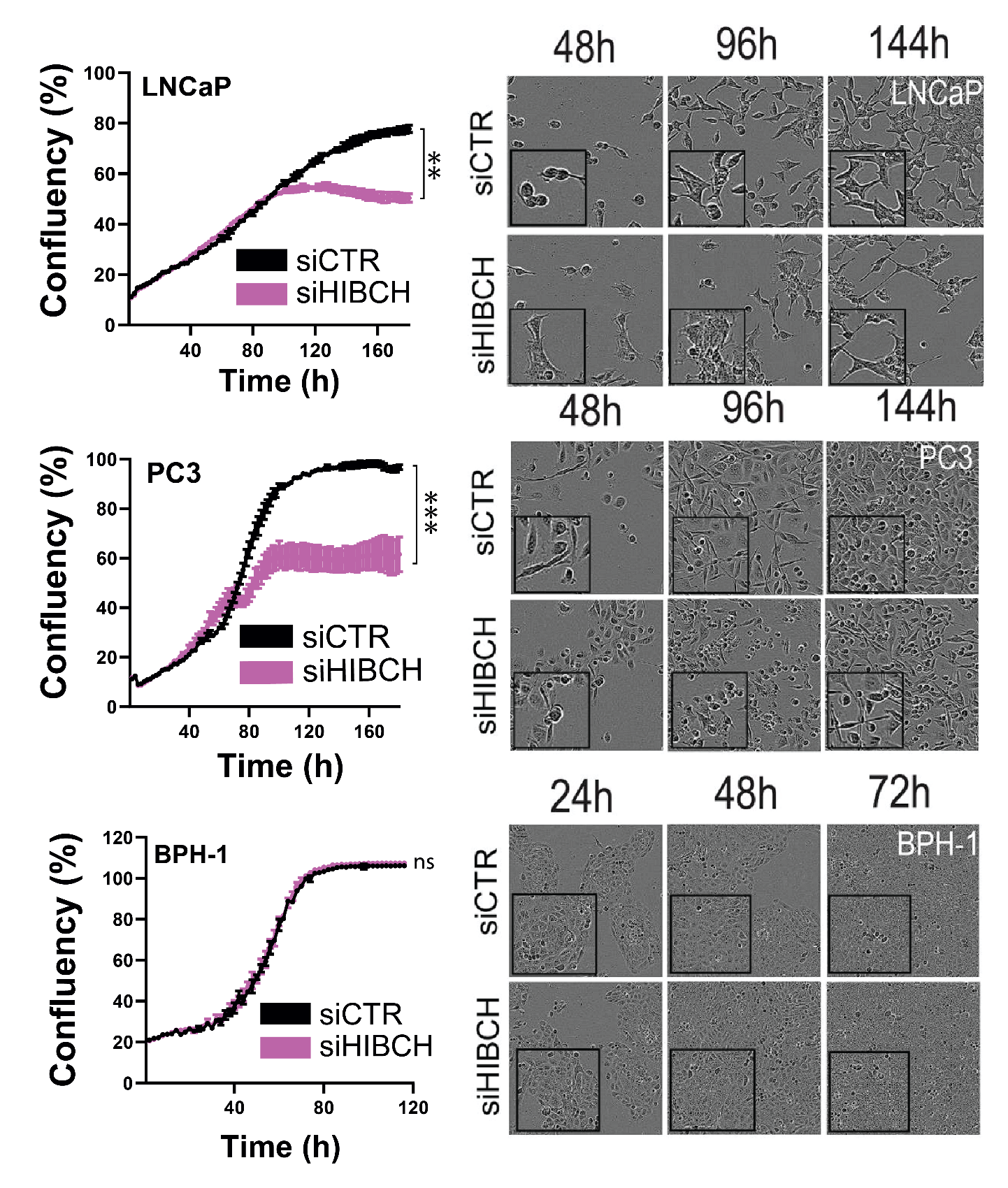


***Supplementary Fig. 4: PCa cell morphology is altered following HIBCH knockdown.***

*Cell confluency time course assay and respective images of LNCaP, PC3 and BPH-1 cells, following siRNA transfection of either siCTR or siHIBCH taken with the IncuCyte S3 Live Cell Analyzer.*


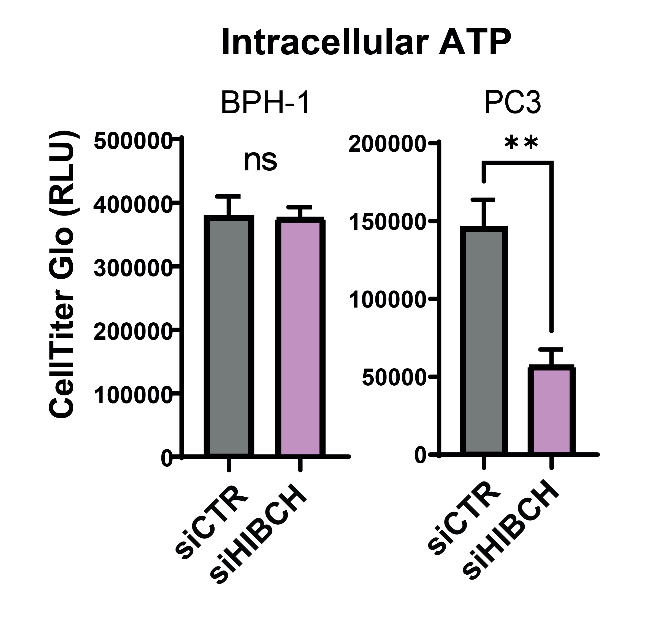


***Supplementary Fig. 5:*** *ATP content of PC3 and BPH-1 cells following 96 hours of siCTR or siHIBCH transfection measured by CellTitre Glo assay.*

***
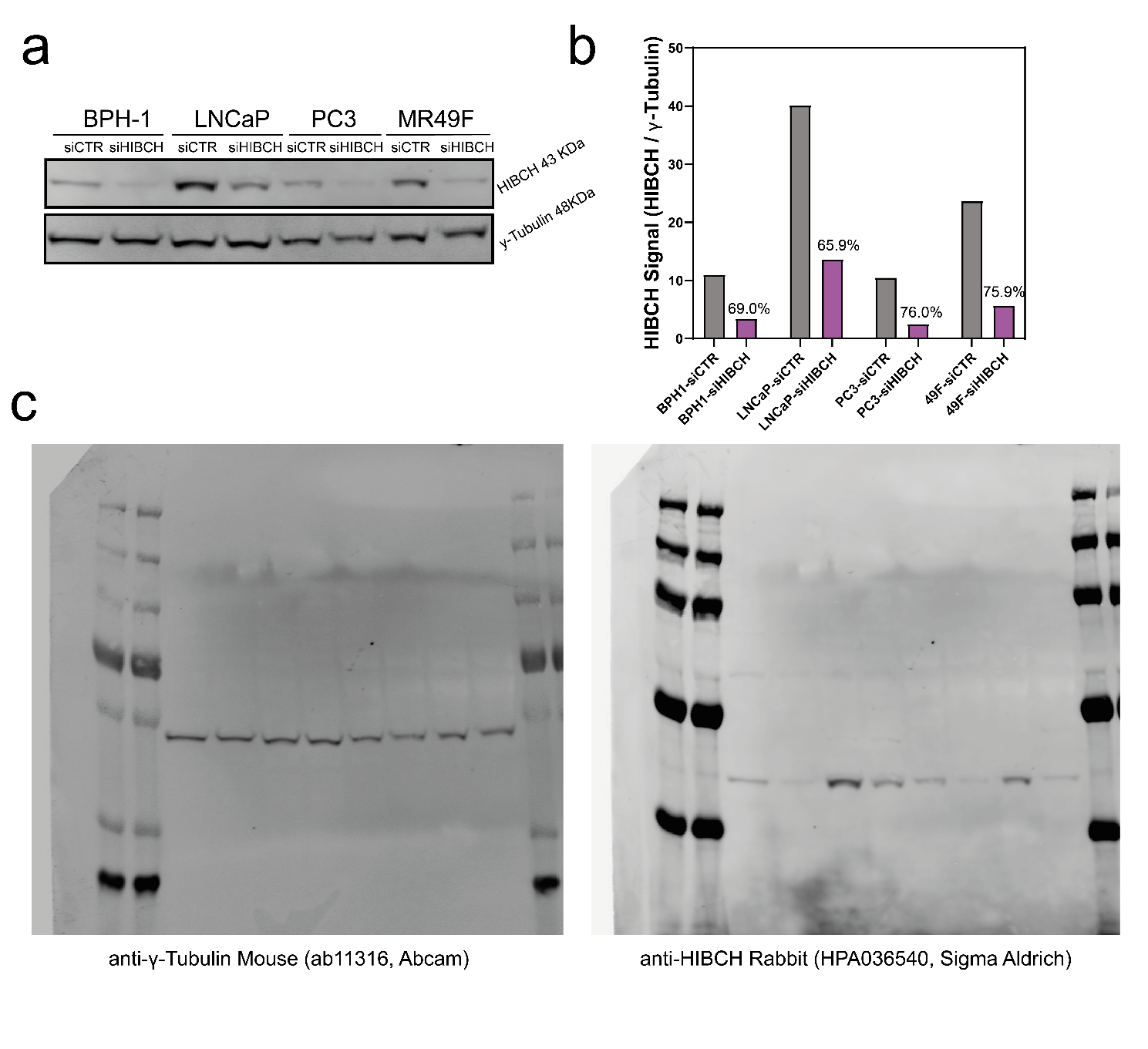
Supplementary Fig. 6: Validation of HIBCH knockdown in LNCaP, PC3, BPH-1 and MR49F cells.***

*(****a****) Western Blot and (****b****) densitometric analysis of HIBCH and y-Tubulin protein in PCa cell lines following 96 hours siRNA (siCTR or siHIBCH) transfection. (****c****) Full western blots with antibody catalogue numbers.*


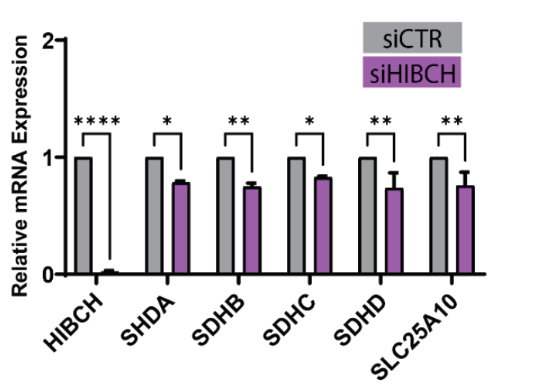


***Supplementary Fig. 7: siRNA mediated suppression of HIBCH reduces succinate-related genes.***

*Gene expression of SDH subunits (A-D) and succinate/malate transporter SLC25A10 at 96 hours post-transfection measured by qRT-PCR analysis.*
