# Supplementary material for: Targeting valine catabolism to inhibit metabolic reprogramming in prostate cancer
Source: Cell Death Dis. 2024 Jul 18;15(7):513. doi: 10.1038/s41419-024-06893-2 (PMC11258138; doi:10.1038/s41419-024-06893-2)
Supplement: Supplementary file 4 — Supplementary Tables 1-6 [file 41419_2024_6893_MOESM4_ESM.docx]

**Targeting Valine Catabolism to Inhibit Metabolic Reprogramming in Prostate Cancer**

Charles L. Bidgood^1†^, Lisa K. Philp^1^, Anja Rockstroh^1^, Melanie Lehman^1,2^, Colleen C. Nelson^1^, Martin C. Sadowski^3^, and Jennifer H. Gunter^1†^

**Supplementary Tables**

***Supplementary Table. 1:*** *Components of Branched Chain Amino Acid Depleted Medium (BDM) with included concentration and catalogue number.*

| Component | Concentration | Catalogue Number |
| --- | --- | --- |
| RPMI 1640 Medium without Glucose, L-Glutamine, Amino acids | 7.4 g/L | MyBioSource, MBS653421 |
| 11.1 mM Glucose Solution | 11.1 mM | ThermoFisher, A2494001 |
| MEM Non-Essential Amino Acids Solution | 1X | ThermoFisher, 11140050 |
| L-Arginine | 1.15 mM | Sigma-Aldrich, A8094 |
| L-Cystine Dihydrochloride | 0.21 mM | Sigma-Aldrich, C6727 |
| L-Glutamine | 2.05 mM | Sigma-Aldrich, G8540 |
| L-Histidine | 0.1 mM | Sigma-Aldrich, H6034 |
| L-Lysine monohydrochloride | 0.27 mM | Sigma-Aldrich, L8662 |
| L-Methionine | 0.1 mM | Sigma-Aldrich, M5308 |
| L-Phenylalanine | 0.09 mM | Sigma-Aldrich, P5482 |
| L-Threonine | 0.17 mM | Sigma-Aldrich, T8441 |
| L-Tryptophan | 0.02 mM | Sigma-Aldrich, T8941 |
| L-Tyrosine | 0.16 mM | Sigma-Aldrich, T8566 |

***Supplementary Table. 2:*** *Forward and reverse primers used for quantitative reverse transcriptase polymerase chain reaction (qRT-PCR) analysis.*

| Gene | Forward Sequence (5’- 3’) | Reverse Sequence (5’- 3’) |
| --- | --- | --- |
| ACAT1 | AATGAACAGAGGATCAACACC | GTGCAATATTCAGCTTCTTTGC |
| ACC1a | CTGTAGAAACCCGGACAGTAGA | GGTCAGCATACATCTCCATGTG |
| BCAT1 | AGAGTTTGGATGGGAGAAACC | GGATCAAGAATGGGTCCCA |
| BCAT2 | GGTGTTTGGGAAGACATTTACC | CGTTCAAAGGCAAAGACCAG |
| GOT2 | GATCCGTCCCATGTATTCC | CCATGACTTTCACTTCTTGC |
| HIBCH | GTTAGAGGAAGATTTGTTAGCC | GTCTCGATCAATCTTAGACTC |
| HMGCR | GGATGACTCGTGGCCCAGT | TCGAGCCAGGCTTTCACTTC |
| HMGCS | TTCACCATGCCTGGATCACTT | ATCTCAAGGGCAACAATTCCC |
| MCCC2 | AGAAAGTCTGGAGTAAGTGAC | CTCTTCAGAAGGTTCAATGG |
| MCEE | GTCGACTCAACCATGTAGCC | CCTTGTGGAACTGGAGCAA |
| MUT | ATTCACACGTGGACCATATCC | CCTTTGATCTGGCGACACA |
| PCCA | AGATGGGCATTAAGACAGTTGC | GATGAGGCTGTCTGTGTTG |
| PCCB | CTCTGTTAACGAACGCATCG | GGATCAGTCTCTTGCTGGAC |
| RPL32 | GCACCAGTCAGACCGATATG | ACTGGGCAGCATGTGCTTTG |
| SDHA | AGCATGCAGAAGTCAATG | ATTTTCCCACAACCTTCTTG |
| SDHB | AGTTCTTATGCAGGCCTATC | GGTATAGAGAGAATGGGTCC |
| SDHC | TATCTACAGTTGGTCTCTTCC | CATAGAGGACAACACAGTAAG |
| SDHD | TATTTGAATCCTTGCTCTGC | TAGTTGAAATAGCAAAGCCC |
| SLC25A10 | AGTATCAGGGCGTTTTCC | CAAAGTTTTTGCGTAGCTG |
| SUCLG2 | TGATCCTAAGGTTGAAGCC | GTTGTTGAGTATCTTCTGGG |

***Supplementary Table. 3:*** *Top 40 co-expressed genes correlated with HIBCH expression in patients with prostate adenocarcinoma. Spearman’s correlation performed on raw data obtained from The Cancer Genome Atlas’ (TCGA) Prostate Adenocarcinoma (TCGA-PRAD) dataset.*

| Gene | Rank | Spearman's | p Value | q Value |
| --- | --- | --- | --- | --- |
| ETFA | 1 | 0.48804 | 1.44E-30 | 2.87E-26 |
| PCCA | 2 | 0.47037 | 3.11E-28 | 3.11E-24 |
| WDR61 | 3 | 0.46159 | 4.02E-27 | 2.68E-23 |
| ERLEC1 | 4 | 0.42332 | 1.22E-22 | 6.12E-19 |
| EMC7 | 5 | 0.41602 | 7.59E-22 | 3.04E-18 |
| SCP2 | 6 | 0.4116 | 2.24E-21 | 6.77E-18 |
| STAMBP | 7 | 0.41137 | 2.37E-21 | 6.77E-18 |
| ALDH9A1 | 8 | 0.41029 | 3.08E-21 | 7.69E-18 |
| TMEM59 | 9 | 0.40532 | 1.02E-20 | 2.26E-17 |
| PPA2 | 10 | 0.39218 | 2.17E-19 | 4.33E-16 |
| MDH1 | 11 | 0.38992 | 3.61E-19 | 6.57E-16 |
| SUCLG2 | 12 | 0.38739 | 6.39E-19 | 1.06E-15 |
| HADHB | 13 | 0.3853 | 1.02E-18 | 1.57E-15 |
| ZSWIM4 | 14 | -0.3815 | 2.38E-18 | 3.40E-15 |
| RPN2 | 15 | 0.38077 | 2.77E-18 | 3.55E-15 |
| C1GALT1C1 | 16 | 0.38065 | 2.84E-18 | 3.55E-15 |
| UQCRFS1 | 17 | 0.37916 | 3.94E-18 | 4.63E-15 |
| SDHC | 18 | 0.37461 | 1.05E-17 | 1.17E-14 |
| MYCBP | 19 | 0.37423 | 1.14E-17 | 1.20E-14 |
| TMED10 | 20 | 0.37379 | 1.25E-17 | 1.25E-14 |
| ARL1 | 21 | 0.37334 | 1.4E-17 | 1.3E-14 |
| SEMA6C | 22 | -0.373 | 1.5E-17 | 1.3E-14 |
| CDK18 | 23 | -0.3722 | 1.8E-17 | 1.5E-14 |
| RAB18 | 24 | 0.37176 | 1.9E-17 | 1.6E-14 |
| ATP5PB | 25 | 0.37171 | 2E-17 | 1.6E-14 |
| ACADSB | 26 | 0.37037 | 2.6E-17 | 2.0E-14 |
| CPT2 | 27 | 0.36987 | 2.9E-17 | 2.1E-14 |
| PDIA3 | 28 | 0.3688 | 3.6E-17 | 2.6E-14 |
| MED31 | 29 | 0.36867 | 3.7E-17 | 2.6E-14 |
| DOK3 | 30 | -0.3682 | 4.1E-17 | 2.7E-14 |
| MPHOSPH6 | 31 | 0.36412 | 9.6E-17 | 6.2E-14 |
| GPHN | 32 | 0.3632 | 1.2E-16 | 7.2E-14 |
| HDHD2 | 33 | 0.36188 | 1.5E-16 | 9.2E-14 |
| ADI1 | 34 | 0.361 | 1.8E-16 | 1.1E-13 |
| PPCS | 35 | 0.36015 | 2.2E-16 | 1.2E-13 |
| OS9 | 36 | 0.35988 | 2.3E-16 | 1.3E-13 |
| OR51E2 | 37 | 0.3577 | 3.6E-16 | 1.9E-13 |
| SELENOF | 38 | 0.35754 | 3.7E-16 | 1.9E-13 |
| NDUFB3 | 39 | 0.35669 | 4.4E-16 | 2.2E-13 |
| MLH1 | 40 | 0.35565 | 5.4E-16 | 2.7E-13 |

***Supplementary Table. 4:*** *Relative metabolite fold change values of shHIBCH LNCaP cells determined by liquid chromatography–mass spectrometry (LCMS). Values were calculated by normalising shNT and shHIBCH cells against their non-induced doxycycline-induced controls. Relative fold change values were then derived from the mean of the non-targeting shRNA control (shNT)*

| Metabolite | HMDB ID | shNT1 | shNT2 | shHIBCH1 | shHIBCH2 | Average FC |
| --- | --- | --- | --- | --- | --- | --- |
| 3-phospho-D-glycerate | HMDB0060180 | 0.95 | 1.05 | 0.74 | 0.74 | 0.74 |
| 6-phosphogluconic acid | HMDB0001316 | 0.99 | 1.01 | 0.93 | 0.93 | 0.93 |
| Aconitate | HMDB0000072 | 0.94 | 1.06 | 0.56 | 0.64 | 0.60 |
| Acetyl coenzyme A | HMDB0001206 | 0.98 | 1.02 | 0.94 | 0.93 | 0.94 |
| Adenosine monophosphate | HMDB0000045 | 0.93 | 1.07 | 0.88 | 0.85 | 0.87 |
| Adenosine triphosphate | HMDB0000538 | 0.93 | 1.07 | 0.58 | 0.63 | 0.61 |
| Citrate | HMDB0000094 | 0.87 | 1.13 | 0.20 | 0.23 | 0.22 |
| Cytidine monophosphate | HMDB0000095 | 0.75 | 1.25 | 0.46 | 0.44 | 0.45 |
| Phosphocreatine | HMDB0001511 | 0.93 | 1.07 | 0.46 | 0.40 | 0.43 |
| Cytidine triphosphate | HMDB0000082 | 0.88 | 1.12 | 0.81 | 0.82 | 0.81 |
| Erythrose 4-phosphate | HMDB0001321 | 1.00 | 1.00 | 0.74 | 0.98 | 0.86 |
| Fructose 1,6-bisphosphate | HMDB0001058 | 0.74 | 1.26 | 0.56 | 0.58 | 0.57 |
| Fructose 6-phosphate | HMDB0000124 | 1.00 | 1.00 | 0.48 | 0.67 | 0.58 |
| Fumarate | HMDB0000134 | 0.984 | 1.016 | 0.574 | 0.499 | 0.54 |
| Glucose 1-phosphate | HMDB0001586 | 1.101 | 0.899 | 0.562 | 0.688 | 0.63 |
| Glycerol-3-phosphate | HMDB0000126 | 0.901 | 1.099 | 0.634 | 0.621 | 0.63 |
| Glucose 6-phosphate | HMDB0001401 | 0.926 | 1.074 | 0.481 | 0.598 | 0.54 |
| Glyceraldehyde 3-phosphate | HMDB0001112 | 1.234 | 0.766 | 0.529 | 0.557 | 0.54 |
| Guanosine diphosphate | HMDB0001201 | 0.940 | 1.060 | 0.734 | 0.734 | 0.73 |
| Glycolic acid | HMDB0000115 | 0.961 | 1.039 | 0.645 | 0.412 | 0.53 |
| Guanosine monophosphate | HMDB0001397 | 0.987 | 1.013 | 0.866 | 0.841 | 0.85 |
| Guanosine triphosphate | HMDB0001273 | 0.940 | 1.060 | 0.606 | 0.633 | 0.62 |
| Isocitrate | HMDB0000193 | 0.987 | 1.013 | 0.903 | 0.908 | 0.91 |
| α-ketoglutarate | HMDB0000208 | 1.006 | 0.994 | 1.059 | 1.049 | 1.05 |
| Lactate | HMDB0000190 | 1.010 | 0.990 | 0.666 | 0.694 | 0.68 |
| Malate | HMDB0000156 | 1.009 | 0.991 | 0.820 | 0.759 | 0.79 |
| NAD+ | HMDB0001179 | 0.928 | 1.072 | 0.522 | 0.654 | 0.59 |
| NADH | HMDB0001487 | 1.010 | 0.990 | 1.001 | 0.998 | 1.00 |
| NADP+ | HMDB0000217 | 0.940 | 1.060 | 0.680 | 0.651 | 0.67 |
| NADPH | HMDB0000221 | 0.923 | 1.077 | 0.733 | 0.705 | 0.72 |
| Phosphoenolpyruvate | HMDB0000263 | 0.914 | 1.086 | 0.415 | 0.353 | 0.38 |
| Ribose 5-phosphate | HMDB0001548 | 0.981 | 1.019 | 0.780 | 0.855 | 0.82 |
| Ribulose 5-phosphate | HMDB0000618 | 1.009 | 0.991 | 0.702 | 0.732 | 0.72 |
| Succinyl-CoA | HMDB0001022 | 0.881 | 1.119 | 0.670 | 0.688 | 0.68 |
| Succinate | HMDB0000254 | 1.042 | 0.958 | 0.613 | 0.603 | 0.61 |
| Uridine diphosphate | HMDB0000295 | 0.682 | 1.318 | 0.684 | 0.704 | 0.69 |
| Uridine diphosphate glucuronic acid | HMDB0000935 | 0.935 | 1.065 | 0.570 | 0.611 | 0.59 |
| Uridine diphosphate N-Acetylglucosamine | HMDB0000290 | 0.856 | 1.144 | 0.395 | 0.476 | 0.44 |
| Uridine monophosphate | HMDB0000288 | 0.542 | 1.458 | 0.240 | 0.365 | 0.30 |
| Xylulose 5-phosphate | HMDB0000868 | 0.983 | 1.017 | 0.710 | 0.710 | 0.71 |

***Supplementary Table. 5:*** *Gene Set Variation Analysis (GSVA) analysis performed on RNA sequencing data of LNCaP cells transfected with siRNAs targeting HIBCH (siHIBCH) or a non-targeting control (siCTR). GSVA was performed on transcript per million (TPM) expression values from each sample and analysed against KEGG metabolic gene signatures obtained from the Molecular Signatures Database (mSigDB).*

| KEGG Pathway Entry | | | | siCTR 1 | siCTR 2 | siHIBCH 1 | siHIBCH 2 |
| --- | --- | --- | --- | --- | --- | --- | --- |
| Galactose Metabolism | | | | 0.43 | 0.36 | -0.61 | -0.11 |
| Fructose And Mannose Metabolism | | | | 0.47 | 0.12 | -0.60 | -0.22 |
| Arachidonic Acid Metabolism | | | | 0.46 | 0.31 | -0.39 | -0.23 |
| Porphyrin And Chlorophyll Metabolism | | | | 0.36 | 0.26 | -0.23 | -0.41 |
| Beta Alanine Metabolism | | | | 0.40 | 0.18 | -0.34 | -0.19 |
| Taurine And Hypotaurine Metabolism | | | | 0.33 | 0.05 | -0.26 | -0.42 |
| Glycolysis Gluconeogenesis | | | | 0.41 | 0.16 | -0.41 | -0.04 |
| Oxidative Phosphorylation | | | | 0.17 | 0.31 | -0.22 | -0.27 |
| Aminoacyl tRNA Biosynthesis | | | | 0.16 | 0.27 | -0.13 | -0.40 |
| Glutathione Metabolism | | | | 0.15 | 0.29 | -0.17 | -0.35 |
| Pentose Phosphate Pathway | | | | 0.30 | 0.20 | -0.54 | 0.10 |
| Glyoxylate And Dicarboxylate Metabolism | | | | 0.24 | 0.31 | -0.10 | -0.29 |
| Regulation Of Autophagy | | | | -0.06 | 0.51 | 0.07 | -0.51 |
| Peroxisome | | | | 0.31 | 0.11 | -0.16 | -0.31 |
| Citrate Cycle TCA Cycle | | | | 0.18 | 0.28 | -0.14 | -0.28 |
| Pyruvate Metabolism | | | | 0.30 | 0.20 | -0.22 | -0.12 |
| Amino Sugar And Nucleotide Sugar Metabolism | | | | 0.12 | 0.21 | -0.27 | -0.19 |
| Ether Lipid Metabolism | | | | 0.22 | 0.24 | -0.21 | -0.08 |
| Fatty Acid Metabolism | | | | 0.31 | 0.02 | -0.25 | -0.14 |
| Valine Leucine and Isoleucine Degradation | | | | -0.02 | 0.28 | -0.19 | -0.24 |
| Glycerolipid Metabolism | | | | 0.11 | 0.27 | -0.25 | -0.03 |
| Starch And Sucrose Metabolism | | | | 0.16 | 0.18 | 0.10 | -0.38 |
| Glycerophospholipid Metabolism | | | | 0.05 | 0.30 | -0.26 | -0.01 |
| Sphingolipid Metabolism | | | | 0.14 | 0.15 | -0.15 | -0.17 |
| Lysosome | | | | 0.12 | 0.18 | -0.30 | 0.01 |
| Retinol Metabolism | | | | 0.36 | -0.11 | -0.19 | -0.10 |
| Propanoate Metabolism | | | | -0.01 | 0.25 | 0.07 | -0.34 |
| Alpha Linolenic Acid Metabolism | | | | 0.40 | -0.05 | -0.35 | 0.19 |
| Phenylalanine Metabolism | | | | 0.35 | 0.00 | -0.27 | 0.12 |
| Butanoate Metabolism | | | | 0.08 | 0.17 | -0.12 | -0.12 |
| Pantothenate And Coa Biosynthesis | | | | -0.07 | 0.48 | -0.11 | 0.04 |
| Selenoamino Acid Metabolism | | | | 0.32 | -0.03 | 0.07 | -0.23 |
| Steroid Hormone Biosynthesis | | | | 0.06 | 0.20 | 0.07 | -0.24 |
| Linoleic Acid Metabolism | | | | 0.28 | -0.08 | -0.25 | 0.06 |
| Pyrimidine Metabolism | | | | 0.01 | 0.11 | -0.09 | -0.13 |
| Terpenoid Backbone Biosynthesis | | | | -0.12 | 0.10 | -0.15 | -0.18 |
| Tyrosine Metabolism | | | | 0.30 | -0.08 | -0.14 | 0.06 |
| O Glycan Biosynthesis | | | | 0.29 | -0.18 | 0.00 | -0.16 |
| Nicotinate And Nicotinamide Metabolism | | | | 0.10 | 0.06 | 0.04 | -0.12 |
| Inositol Phosphate Metabolism | | | | -0.03 | 0.11 | -0.12 | 0.00 |
| Purine Metabolism | | | | 0.04 | 0.06 | -0.08 | -0.02 |
| Adipocytokine Signaling Pathway | | | | 0.30 | -0.21 | -0.17 | 0.07 |
| Cysteine And Methionine Metabolism | | | | 0.06 | -0.03 | 0.08 | -0.23 |
| N Glycan Biosynthesis | | | | -0.05 | 0.15 | -0.08 | 0.02 |
| Glycosaminoglycan Biosynthesis Keratan Sulfate | | | | 0.23 | -0.14 | -0.24 | 0.17 |
| Histidine Metabolism | | | | 0.21 | -0.04 | 0.06 | 0.00 |
| ABC Transporters | | | | 0.14 | -0.11 | -0.13 | 0.07 |
| Sulphur Metabolism | | | | 0.35 | -0.30 | -0.04 | 0.07 |
| Glycosylphosphatidylinositol GPI Anchor Biosynthesis | | | | 0.00 | 0.08 | 0.00 | 0.04 |
| One Carbon Pool by Folate | | | | 0.23 | -0.14 | 0.29 | -0.22 |
| Riboflavin Metabolism | | | | -0.17 | 0.13 | -0.09 | 0.05 |
| Pentose And Glucuronate Interconversions | | | | -0.26 | 0.29 | 0.24 | -0.21 |
| Ascorbate And Aldarate Metabolism | | | | -0.17 | 0.15 | 0.20 | -0.22 |
|  | | | |  |  |  |  |
|  | | | |  |  |  |  |
|  |  |  |  |  |  |  |  |
| *Supplementary Table. 5 (Continued)* | | | |  |  |  |  |
| KEGG Pathway Entry | | | | siCTR 1 | siCTR 2 | siHIBCH 1 | siHIBCH 2 |
| … | | | |  |  |  |  |
| mTOR Signaling Pathway | | | | -0.12 | 0.11 | -0.07 | 0.09 |
| Insulin Signaling Pathway | | | | 0.02 | -0.10 | -0.11 | 0.21 |
| Arginine And Proline Metabolism | | | | 0.15 | -0.28 | -0.22 | 0.29 |
| Steroid Biosynthesis | | | | -0.01 | -0.05 | 0.20 | -0.05 |
| Primary Bile Acid Biosynthesis | | | | -0.13 | -0.11 | 0.17 | -0.14 |
| Alanine Aspartate and Glutamate Metabolism | | | | -0.07 | -0.15 | -0.13 | 0.28 |
| Tryptophan Metabolism | | | | -0.29 | -0.08 | -0.12 | 0.24 |
| Limonene And Pinene Degradation | | | | -0.24 | -0.19 | -0.11 | 0.19 |
| Glycine Serine and Threonine Metabolism | | | | 0.14 | -0.45 | -0.08 | 0.31 |
| Biosynthesis Of Unsaturated Fatty Acids | | | | -0.17 | -0.28 | 0.00 | 0.10 |
| Lysine Degradation | | | | -0.13 | -0.17 | 0.20 | 0.13 |
| Glycosaminoglycan Degradation | | | | -0.51 | -0.01 | -0.19 | 0.31 |
| Folate Biosynthesis | | | | -0.18 | -0.36 | 0.15 | 0.29 |
| Nitrogen Metabolism | | | | -0.45 | -0.30 | 0.36 | 0.23 |

***Supplementary Table. 6:*** *MetaboAnalyst (5.0) Joint Pathway Analysis performed on RNA sequencing and metabolomics data. Total. Total – total combined pathway entries, FDR - False Discovery Rate, Holm – Holm Adjusted p value, Impact – impact score.*

| Pathway | Total | Expected | Hits | Raw p | -log(p) | Holm | FDR | Impact |
| --- | --- | --- | --- | --- | --- | --- | --- | --- |
| Ascorbate and aldarate metabolism | 13 | 0.50 | 8 | 3.6E-09 | 8.44 | 3.1E-07 | 3.1E-07 | 0.67 |
| Citrate cycle (TCA cycle) | 42 | 1.60 | 11 | 2.2E-07 | 6.66 | 1.8E-05 | 6.3E-06 | 1.27 |
| Glycolysis or Gluconeogenesis | 61 | 2.33 | 13 | 2.3E-07 | 6.65 | 1.8E-05 | 6.3E-06 | 0.72 |
| Pentose and glucuronate interconversions | 32 | 1.22 | 9 | 1.6E-06 | 5.81 | 1.3E-04 | 3.3E-05 | 0.48 |
| Pentose phosphate pathway | 47 | 1.79 | 9 | 4.7E-05 | 4.32 | 3.8E-03 | 8.0E-04 | 1.26 |
| Pyruvate metabolism | 45 | 1.72 | 8 | 2.2E-04 | 3.65 | 1.8E-02 | 3.1E-03 | 0.68 |
| Purine metabolism | 166 | 6.33 | 15 | 1.2E-03 | 2.93 | 9.3E-02 | 3.1E-03 | 0.59 |
| Retinol metabolism | 47 | 1.79 | 7 | 1.7E-03 | 2.77 | 1.3E-01 | 3.1E-03 | 0.30 |
| Butanoate metabolism | 29 | 1.11 | 5 | 4.1E-03 | 2.38 | 3.2E-01 | 3.1E-03 | 0.43 |
| Pyrimidine metabolism | 99 | 3.77 | 9 | 1.2E-02 | 1.93 | 8.8E-01 | 3.1E-03 | 0.42 |
| Porphyrin and chlorophyll metabolism | 53 | 2.02 | 6 | 1.4E-02 | 1.85 | 1.0E+00 | 3.1E-03 | 0.08 |
| Drug metabolism - other enzymes | 70 | 2.67 | 7 | 1.6E-02 | 1.80 | 1.0E+00 | 3.1E-03 | 0.07 |
| Arginine biosynthesis | 27 | 1.03 | 4 | 1.8E-02 | 1.75 | 1.0E+00 | 3.1E-03 | 0.27 |
| Glyoxylate and dicarboxylate metabolism | 56 | 2.13 | 6 | 1.8E-02 | 1.74 | 1.0E+00 | 3.1E-03 | 0.27 |
| Starch and sucrose metabolism | 43 | 1.64 | 5 | 2.2E-02 | 1.66 | 1.0E+00 | 3.1E-03 | 0.48 |
| beta-Alanine metabolism | 44 | 1.68 | 5 | 2.4E-02 | 1.62 | 1.0E+00 | 3.1E-03 | 0.23 |
| Alanine, aspartate and glutamate metabolism | 61 | 2.33 | 6 | 2.7E-02 | 1.57 | 1.0E+00 | 3.1E-03 | 0.22 |
| Drug metabolism - cytochrome P450 | 98 | 3.74 | 8 | 3.1E-02 | 1.51 | 1.0E+00 | 3.1E-03 | 0.14 |
| Synthesis and degradation of ketone bodies | 10 | 0.38 | 2 | 5.3E-02 | 1.28 | 1.0E+00 | 3.1E-03 | 0.89 |
| Glutathione metabolism | 56 | 2.13 | 5 | 6.0E-02 | 1.22 | 1.0E+00 | 3.1E-03 | 0.29 |
| Propanoate metabolism | 48 | 1.83 | 4 | 1.1E-01 | 0.97 | 1.0E+00 | 3.1E-03 | 0.36 |
| Lysine degradation | 49 | 1.87 | 4 | 1.1E-01 | 0.94 | 1.0E+00 | 3.1E-03 | 0.23 |
| Inositol phosphate metabolism | 69 | 2.63 | 5 | 1.2E-01 | 0.92 | 1.0E+00 | 3.1E-03 | 0.13 |
| Histidine metabolism | 32 | 1.22 | 3 | 1.2E-01 | 0.92 | 1.0E+00 | 3.1E-03 | 0.13 |
| Linoleic acid metabolism | 17 | 0.65 | 2 | 1.4E-01 | 0.87 | 1.0E+00 | 3.1E-03 | 0.13 |
| Neomycin, kanamycin and gentamicin biosynthesis | 4 | 0.15 | 1 | 1.4E-01 | 0.84 | 1.0E+00 | 3.1E-03 | 0.67 |
| Glycerolipid metabolism | 35 | 1.33 | 3 | 1.5E-01 | 0.83 | 1.0E+00 | 3.1E-03 | 0.18 |
| Amino sugar and nucleotide sugar metabolism | 79 | 3.01 | 5 | 1.8E-01 | 0.74 | 1.0E+00 | 3.1E-03 | 0.23 |
| Ether lipid metabolism | 39 | 1.49 | 3 | 1.8E-01 | 0.74 | 1.0E+00 | 3.1E-03 | 0.16 |
| Phenylalanine metabolism | 21 | 0.80 | 2 | 1.9E-01 | 0.72 | 1.0E+00 | 3.1E-03 | 0.40 |
| alpha-Linolenic acid metabolism | 22 | 0.84 | 2 | 2.0E-01 | 0.69 | 1.0E+00 | 3.1E-03 | 0.10 |
| Mucin type O-glycan biosynthesis | 22 | 0.84 | 2 | 2.0E-01 | 0.69 | 1.0E+00 | 3.1E-03 | 0.33 |
| Valine, leucine and isoleucine degradation | 88 | 3.35 | 5 | 2.4E-01 | 0.62 | 1.0E+00 | 3.1E-03 | 0.11 |
| Cysteine and methionine metabolism | 71 | 2.71 | 4 | 2.8E-01 | 0.55 | 1.0E+00 | 3.1E-03 | 0.23 |
| Galactose metabolism | 51 | 1.94 | 3 | 3.1E-01 | 0.51 | 1.0E+00 | 3.1E-03 | 0.20 |
| Metabolism of xenobiotics by cytochrome P450 | 145 | 5.53 | 7 | 3.1E-01 | 0.50 | 1.0E+00 | 3.1E-03 | 0.06 |
| D-Glutamine and D-glutamate metabolism | 10 | 0.38 | 1 | 3.2E-01 | 0.49 | 1.0E+00 | 3.1E-03 | 0.22 |
| Nitrogen metabolism | 10 | 0.38 | 1 | 3.2E-01 | 0.49 | 1.0E+00 | 3.1E-03 | 0.22 |
| Arginine and proline metabolism | 78 | 2.97 | 4 | 3.5E-01 | 0.46 | 1.0E+00 | 3.1E-03 | 0.09 |
| Fatty acid degradation | 102 | 3.89 | 5 | 3.5E-01 | 0.46 | 1.0E+00 | 3.1E-03 | 0.28 |
| Phenylalanine, tyrosine and tryptophan biosynthesis | 11 | 0.42 | 1 | 3.5E-01 | 0.46 | 1.0E+00 | 3.1E-03 | 0.80 |
| Sphingolipid metabolism | 58 | 2.21 | 3 | 3.8E-01 | 0.42 | 1.0E+00 | 3.1E-03 | 0.16 |
| Terpenoid backbone biosynthesis | 36 | 1.37 | 2 | 4.0E-01 | 0.40 | 1.0E+00 | 3.1E-03 | 0.14 |
| Thiamine metabolism | 14 | 0.53 | 1 | 4.2E-01 | 0.38 | 1.0E+00 | 3.1E-03 | 0.15 |
| Tyrosine metabolism | 88 | 3.35 | 4 | 4.3E-01 | 0.36 | 1.0E+00 | 3.1E-03 | 0.30 |
| Lipoic acid metabolism | 15 | 0.57 | 1 | 4.4E-01 | 0.35 | 1.0E+00 | 3.1E-03 | 0.14 |
| Fructose and mannose metabolism | 40 | 1.52 | 2 | 4.6E-01 | 0.34 | 1.0E+00 | 3.1E-03 | 0.23 |
| Nicotinate and nicotinamide metabolism | 42 | 1.60 | 2 | 4.8E-01 | 0.32 | 1.0E+00 | 3.1E-03 | 0.27 |
| Sulfur metabolism | 18 | 0.69 | 1 | 5.0E-01 | 0.30 | 1.0E+00 | 3.1E-03 | 0.12 |
| Steroid biosynthesis | 82 | 3.13 | 3 | 6.1E-01 | 0.21 | 1.0E+00 | 3.1E-03 | 0.47 |
| Tryptophan metabolism | 84 | 3.20 | 3 | 6.3E-01 | 0.20 | 1.0E+00 | 3.1E-03 | 0.06 |
| Glycerophospholipid metabolism | 86 | 3.28 | 3 | 6.5E-01 | 0.19 | 1.0E+00 | 3.1E-03 | 0.14 |
| Mannose type O-glycan biosynthesis | 30 | 1.14 | 1 | 6.9E-01 | 0.16 | 1.0E+00 | 3.1E-03 | 0.03 |
| Pantothenate and CoA biosynthesis | 34 | 1.30 | 1 | 7.4E-01 | 0.13 | 1.0E+00 | 3.1E-03 | 0.18 |
| Phosphatidylinositol signaling system | 74 | 2.82 | 2 | 7.8E-01 | 0.11 | 1.0E+00 | 3.1E-03 | 0.14 |
| Steroid hormone biosynthesis | 199 | 7.58 | 6 | 7.8E-01 | 0.11 | 1.0E+00 | 3.1E-03 | 0.11 |
| Fatty acid elongation | 75 | 2.86 | 2 | 7.9E-01 | 0.10 | 1.0E+00 | 3.1E-03 | 0.14 |
| Arachidonic acid metabolism | 81 | 3.09 | 2 | 8.2E-01 | 0.08 | 1.0E+00 | 3.1E-03 | 0.05 |
| Primary bile acid biosynthesis | 92 | 3.51 | 2 | 8.7E-01 | 0.06 | 1.0E+00 | 3.1E-03 | 0.07 |
| Folate biosynthesis | 61 | 2.33 | 1 | 9.1E-01 | 0.04 | 1.0E+00 | 3.1E-03 | 0.03 |
| Glycine, serine and threonine metabolism | 68 | 2.59 | 1 | 9.3E-01 | 0.03 | 1.0E+00 | 3.1E-03 | 0.04 |
| N-Glycan biosynthesis | 77 | 2.93 | 1 | 9.5E-01 | 0.02 | 1.0E+00 | 3.1E-03 | 0.03 |
| Fatty acid biosynthesis | 129 | 4.92 | 1 | 9.9E-01 | 0.00 | 1.0E+00 | 3.1E-03 | 0.08 |
